# Light-Induced Persistent Electronic Chirality in Achiral Molecules Probed with Time-Resolved Electronic Circular Dichroism Spectroscopy

**DOI:** 10.1021/acs.jpclett.5c01808

**Published:** 2025-08-28

**Authors:** Torsha Moitra, Lukas Konecny, Marius Kadek, Ofer Neufeld, Angel Rubio, Michal Repisky

**Affiliations:** † Hylleraas Centre for Quantum Molecular Sciences, Department of Chemistry, 8016 UiT The Arctic University of Norway, 9037 Tromsø, Norway; ‡ Department of Physical and Theoretical Chemistry, Faculty of Natural Sciences, Comenius University, 84215 Bratislava, Slovakia; ¶ Department of Inorganic Chemistry, Faculty of Natural Sciences, Comenius University, 84215 Bratislava, Slovakia; § 375070 Max Planck Institute for the Structure and Dynamics of Matter, Center for Free Electron Laser Science, Luruper Chaussee 149, 22761 Hamburg, Germany; ∥ 26747 Technion Israel Institute of Technology, Faculty of Chemistry, Haifa 3200003, Israel; ⊥ Initiative for Computational Catalysis (ICC), The Flatiron Institute, 162 Fifth Avenue, New York, New York 10010, United States

## Abstract

Chiral systems exhibit unique properties traditionally linked to their asymmetric spatial arrangement. Recently, multiple laser pulses were shown to induce purely electronic chiral states without altering the nuclear configuration. Here, we propose and numerically demonstrate a simpler realization of light-induced electronic chirality that is long-lived and occurs well before the onset of nuclear motion and decoherence. A single monochromatic circularly polarized laser pulse is shown to induce electronic chiral currents in an oriented achiral molecule. Using state-of-the-art ab initio theory, we analyze this effect and relate the chiral currents to induced magnetic dipole moments, detectable via attosecond time-resolved electronic circular dichroism (TR-ECD) spectroscopy, also known as transient absorption ECD. The resulting chiral electronic wavepacket oscillates rapidly in handedness at harmonics of the pump laser’s carrier frequency, and the currents persist after the pulse ends. We establish a chiral molecular-current analogue to high harmonic generation and demonstrate attosecond transient chirality control with potential impact on spintronics and reaction dynamics.

## Graphical abstract

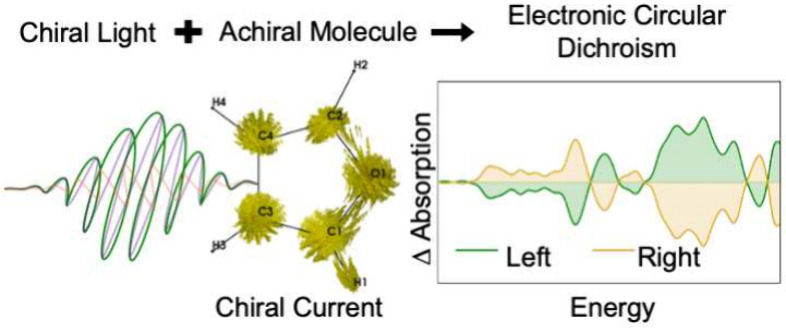

Chirality is a ubiquitous phenomenon observed in nature associated with the lack of mirror-image superimposability of systems ranging from macroscopic objects to molecules.^1,2^ Traditionally, this concept is linked to geometric chirality, where the spatial arrangement of atoms within a molecule creates distinct left and right handedness. Light-induced *nuclear* dynamics operating on femtosecond time scales has shed light on intriguing phenomena where achiral molecules dynamically evolve into geometrically chiral structures via the loss of molecular symmetry.^3–9^ However, it was only recently that the concept of chirality has been realized without the intervention of nuclear degrees of freedom by pure *electronic* motion, giving rise to molecular light-induced electronic chirality.^10,11^ Closely intertwined with the concept of electronic chirality are ring currents, which are known to be induced by chiral light and light with orbital angular momentum.^12^ Helical ring-currents can circulate through molecules^13–18^ or outside of the molecule,^19^ breaking all spatial mirror/inversion symmetries of the system in 3D.^20–22^ In previous works, symmetry breaking has been achieved by using two or more laser pulses, which is intuitively required in order to excite the molecule in a 3D spatial arrangement that breaks all relevant symmetries.^4,11,23,24^

Experimentally, ultrafast chiral states have been measured on the femtosecond time scale using time-resolved photoemission in atoms^16^ and time-resolved photoelectron circular dichroism in chiral molecules.^7,10^ However, to our knowledge, no work to date investigated pure attosecond chiral electron dynamics triggered by a single monochromatic light pulse without ionizing the molecule or breaking it apart. Such studies have been performed for magnetism in solids,^25,26^ but it remains unclear if it is possible to connect attosecond circular-dichroic absorption spectra to chiral currents in molecules. Novel schemes for chiral state control could enable wide-ranging applications such as electronic switches,^27^ spintronics,^28,29^ and phase transitions.^30^

In this Letter, we study light-induced chirality originating from pure electron dynamics in a structurally achiral furan molecule. We show that a single monochromatic circularly polarized pulse is capable of inducing a chiral nonstationary electronic wavepacket in an oriented sample. This effect can be attributed to the induced ring currents generated by the pump pulse, which evolve dynamically in 3D. Interestingly, the current density is long-lived and the nonstationary chiral state does not instantaneously relax to an achiral state, therefore allowing its detection and monitoring and suggesting a way to generate chiral states that should survive up to a few hundred femtoseconds before dephasing. We predict the spectroscopic signature of these chiral wavepackets based on the induced magnetic dipole moments by time-resolved electronic circular dichroism (TR-ECD), also termed transient absorption circular dichroism spectroscopy. These moments rapidly oscillate with frequencies corresponding not only to the pump laser’s carrier frequency but also to its higher-order harmonics that survive long after the laser and permits attosecond transient chiral states. Our work should pave the way to attosecond chiral state manipulation and readout.

This study focuses on the furan molecule, which (i) exhibits an achiral ground-state electronic configuration, (ii) exhibits a static electric dipole moment, which makes it orientable,^31^ and (iii) is frequently used in studies of other ultrafast phenomena.^32–36^ The Supporting Information extends this study to benzene and aniline molecules. Figure 1a illustrates our proposed setup for inducing chiral electron dynamics, where the furan molecule is oriented such that its static electric dipole moment is aligned with the direction of propagation of the CP light (along *z*). The molecule is initially pumped by a chiral, CP left (L) or right (R) laser pulse, whose electric field $E^{L/R}\left(t\right)$ traces a circular trajectory in the *xy* plane

$$E^{L/R}\left(t\right)=E_{0}g\left(t;t_{0},T\right)\left[\cos\left(\omega_{0}\left(t-t_{0}\right)\right)x\mp \sin\left(\omega_{0}\left(t-t_{0}\right)\right)y\right]$$ 1

where the negative and positive combinations correspond to left (L) and right (R) circularly polarized light, respectively. Here, *g*(*t*; *t*_0_, *T*) is a dimensionless Gaussian envelope centered at *t*_0_ and duration *T*, while $E_{0}$ and *ω*_0_ are the amplitude and carrier frequency of the monochromatic light pulse, respectively. Additional details about the pump pulse are given in Section S2. The carrier frequency was tuned to the first bright electronic transition at energy *ℏω*_0_ = 0.223 au = 6.07 eV, while *T* was set to 4.09 fs so that the pump pulse populates primarily the first excited state but also encompasses the second excited state at energy *ℏω*_1_ = 0.294 au = 7.99 eV. A pump pulse amplitude of $E_{0}=0.03$ au corresponding to a peak intensity of 3.16 × 10^13^ W/cm^2^ is used, which leads to about 3% ground state depopulation at the end of the pump, as shown in Section S3.

**Figure 1:**
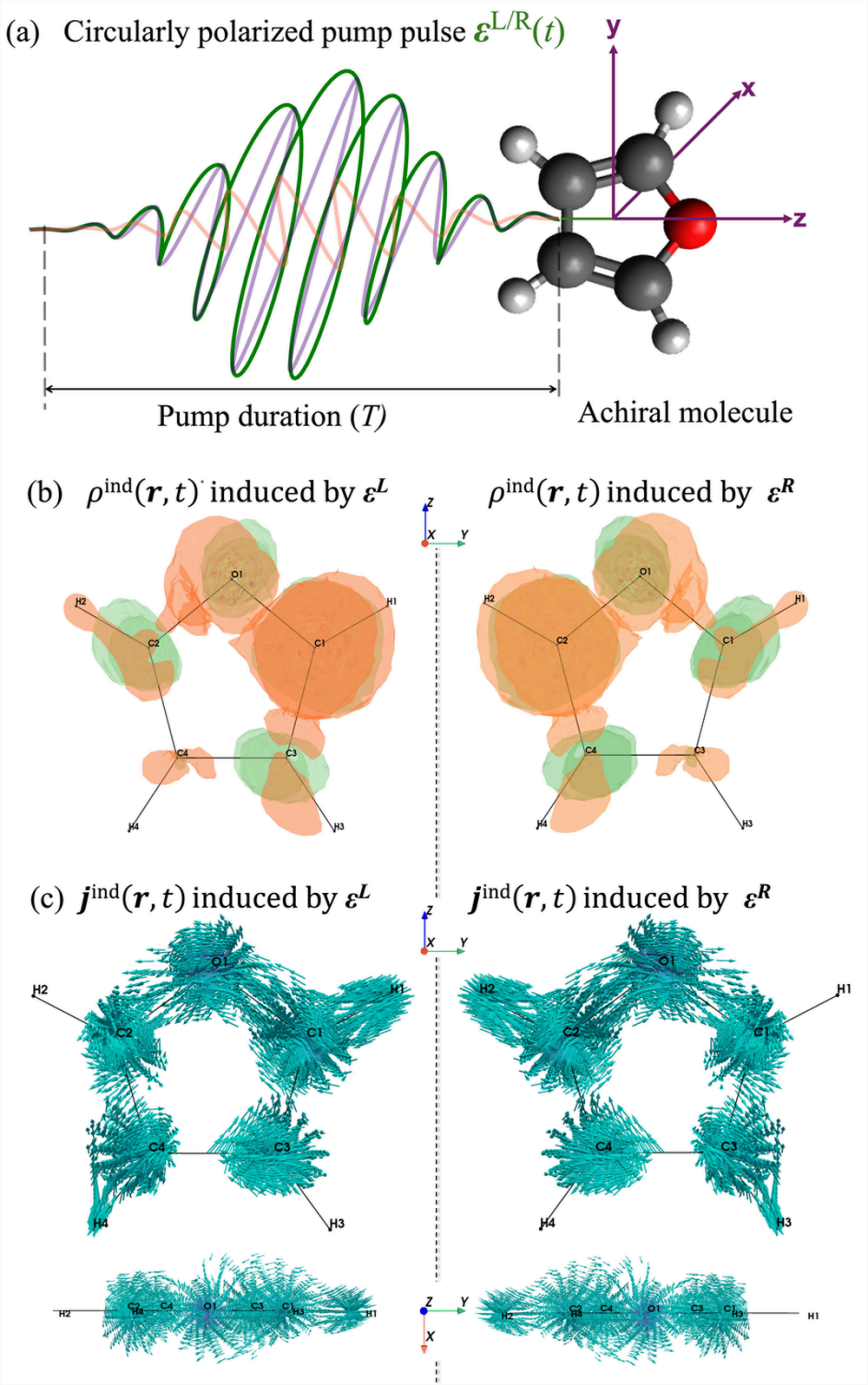
(a) Schematic representation of a circularly polarized pump pulse $E^{L/R}\left(t\right)$ in green with *xy* plane of polarization and *z* propagation, interacting with the achiral furan molecule in *yz* plane. The chiral induced (b) electron charge density *ρ* ^ind^(***r***, *t*) and (c) current density ***j*** ^ind^(***r***, *t*) generated by the circularly polarized light at the end of the pump pulse (*t* = *T*). Gain and loss of charge density are shown by green and orange color surfaces with isovalue 0.005, respectively.

The interaction of an external pulse(s) with the molecule is described from first-principles by real-time time-dependent density functional theory (RT-TDDFT) as implemented in the ReSpect program.^37,38^ The electronic wavepacket is evolved in time domain as per the Liouville-von Neumann (LvN) equation of motion

$$i\frac{\partial D\left(t\right)}{\partial t}=\left[F\left(t\right),D\left(t\right)\right]$$ 2

where **D**(*t*) and **F**(*t*) are the time-dependent one-electron reduced density matrix and the Fock matrix, respectively. **D**(*t*) represents the state of the system, whereas **F**(*t*) characterizes the molecular system and its interaction with the external electric field within the dipole approximation. Both matrices are represented in molecular orbitals (MOs), where each MO is expressed as a linear combination of Gaussian-type orbitals (GTOs). Here, we employed uncontracted aug-cc-pVTZ^39,40^ GTOs and the PBE0 exchange-correlation functional.^41^ We refer the reader to Section S1 for details on the RT-TDDFT methodology.

In order to get insights into ultrafast electron dynamics we compute the time-dependent induced electron charge (*ρ* ^ind^) and current (***j*** ^ind^) densities generated by the external pump pulse $E^{L/R}\left(t\right)$ . In our formalism, these quantities are defined over ground-state MOs (*ϕ*) as

$$\rho^{\mathrm{ind}}\left(r,t\right)=\mathrm{Tr}\left[D^{\mathrm{ind}}\left(t\right)\Omega \left(r\right)\right]$$ 3

$$j_{k}^{\mathrm{ind}}\left(r,t\right)=\mathrm{Tr}\left[D^{\mathrm{ind}}\left(t\right)J_{k}\left(r\right)\right],,k\in x,y,z$$ 4

where **Ω** and ***J***_*k*_ are matrices of the charge density and current density operators

$$\Omega_{pq}\left(r\right)=\phi_{p}^{†}\left(r\right)\phi_{q}\left(r\right)$$ 5

$$J_{k,pq}\left(r\right)=-\frac{1}{2}\left(\phi_{p}^{†}\left(r\right)\left\{p_{k}\phi_{q}\left(r\right)\right\}+\{p_{k}\phi_{p}\left(r\right){\}}^{†}\phi_{q}\left(r\right)\right)$$ 6

The induced density matrix **D** ^ind^(*t*) is obtained as the difference between **D**(*t*) and the static ground-state **D**(0), which evolves on attosecond timescales.

The CPL and CPR pump pulses generate mirror-imaged induced charge and current densities, mimicking molecular enantiomers, as shown in Figures 1b and 1c, respectively. The induced current density has both in-plane and out-of-plane contributions, as shown by the top and side views in Figure 1c. See the Supporting Information for a time-evolution video of the induced charge and current densities. Most importantly, the induced current density gives rise to a corresponding induced magnetic dipole moment in the system, evaluated as

$$m^{\mathrm{ind}}\left(t\right)=\frac{1}{2}\int d^{3}rr\times j^{\mathrm{ind}}\left(r,t\right)$$ 7

Figure 2a displays all components of the induced magnetic moment generated by CPL and CPR pump pulses in green and yellow, respectively. Notably, these induced moments persist well beyond the end of the pump pulse—indicated by the black dashed line in Figure 2a—and exhibit periodic sign reversals. Furthermore, the overall magnitude of the induced magnetic moment | ***m*** ^ind^(*t*)| remains identical for both laser polarizations. A more detailed inspection of the individual components shows that *m*_*x*_^ind^(*t*) and *m*_*z*_^ind^(*t*) have opposite signs for CPL and CPR induced wavepackets, while *m*_*y*_^ind^(*t*) has the same sign for both. This enantiomeric relationship is preserved during and after the pump pulse. Given the relative orientation of our pump laser with respect to the furan molecule, *m*_*x*_^ind^(*t*) has the largest magnitude and can be attributed to the out-of-plane enantiomeric dynamics governed by π-bonding orbitals. A similar enantiomeric behavior is also observed for the in-plane *m*_*z*_^ind^(*t*) component but with lower magnitude. It is important to note that the alternating ring currents and magnetic moments discussed here are induced by a single circularly polarized laser pulse that excites at least two non-degenerate states, as demonstrated in Section S2. In contrast, for molecules belonging to non-Abelian point groups, which possess sets of degenerate states, such laser pulses can induce unidirectional electronic ring currents without any reversals,^13–15^ provided that the carrier frequencies of the laser pulses are resonant with the excitations of selected doubly degenerate states.

**Figure 2:**
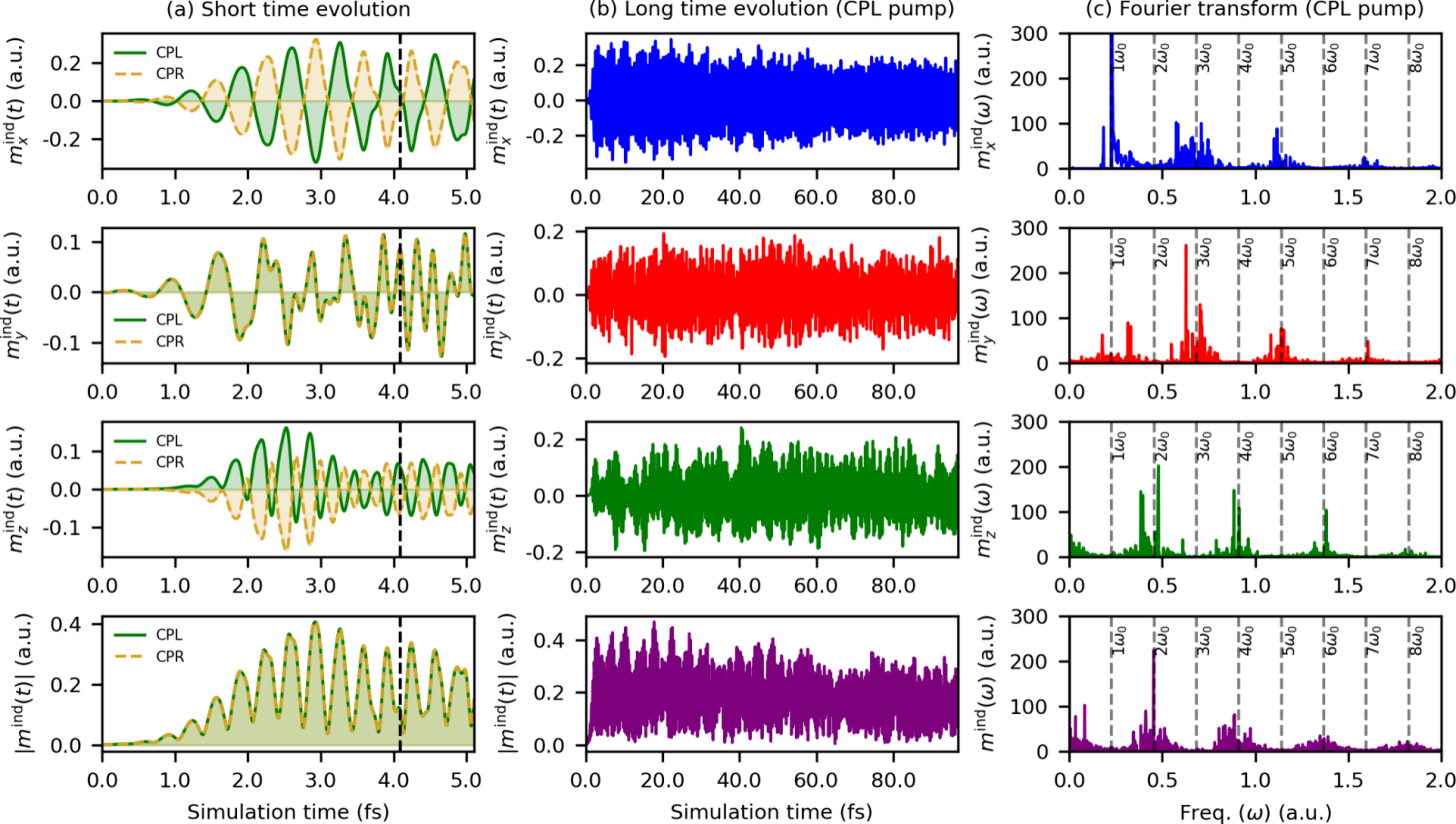
Short time evolution (a) and long time evolution (b) of the magnitude (| ***m*** ^ind^(*t*)|) and components of the induced magnetic dipole moment ***m*** ^ind^(*t*) = (*m*_*x*_^ind^(*t*), *m*_*y*_^ind^(*t*), *m*_*z*_^ind^(*t*)) by CPL and/or CPR pump pulses. The black dashed line in (a) at 4.09 fs marks the end of the pump pulse, after which the electronic wavepacket evolves freely. (c) Fourier transform of the induced magnetic moment obtained from the long time evolution in (b). The harmonic orders of carrier frequency *ω*_0_ = 0.223 au are marked by dashed lines. Note that the simulation does not include dissipative processes.

Figure 2b presents the oscillations in the induced magnetic moment for the CPL induced wavepacket propagated for a long time scale of up to 100 fs. We would like to stress here that our simulations incorporate only pure electron dynamics and have no external decoherence effects (nuclear motion, solvent effect, etc.), which would gain prominence at such large timescales.^42^ Nevertheless, even in this idealized scenario, the oscillations of the induced magnetic moment do not exhibit a single dominant frequency. A Fourier transform of the time-domain signal shows a contribution from multiple harmonic orders of the laser pulse carrier frequency (*ω*_0_), marked by dashed lines in Figure 2c. Therefore, it is impossible to define a single characteristic frequency for the flipping of the chiral state from one form to the other. The presence of higher harmonics also indicates that the induced electronic chirality opens up a route to coherent manipulation of ultrafast chiral states. The observation of multiple harmonic orders in the induced magnetic dipole moment suggests a light–matter mechanism analogous to high harmonic generation (HHG)^43–45^ but where dynamics involved multiple harmonics even in much longer timescales, and in molecular magnetic moments rather than the typical dipole response that emits photons. The observed higher-order harmonics in the chiral current tune the speed with which the molecular handedness can ultimately be controlled, and here they should be interpreted solely as signatures of intense nonlinear responses rather than molecular ionizations. Moreover, the observation that even harmonic orders appear in *m*_z_^ind^(*ω*) while odd harmonic orders are present in *m*_*x*_^ind^(*ω*) and *m*_y_^ind^(*ω*) suggests a symmetry-guided selection rule at play, possibly due to the parity of the chiral wavepacket.^24^ The corresponding induced electric dipole moment and its Fourier transform are shown in Section S4. This rich harmonic content provides a spectral fingerprint of the underlying chiral dynamics and highlights the complex interplay between electronic motion and the system’s symmetry. Overall, these results suggest a route toward obtaining attosecond chiral transients even in the absence of using attosecond circularly polarized pulses, which are very difficult to generate experimentally.^46^

Having established the generation of chiral persistent currents with attosecond characteristics, let us discuss their potential observation. A standard approach to spectroscopically detect the induced magnetic dipole moment in structurally chiral systems is through electronic circular dichroism (ECD) absorption spectroscopy, which measures the differential absorption of left and right circularly polarized light.^47–51^ We extend this technique to a typical pump–probe setup for obtaining a time-resolved (TR-)­ECD spectral signature, where the pump pulse, as discussed above, induces the electronic chirality in achiral oriented furan, while the probe pulse spectroscopically measures the induced chirality. This methodology forms the foundation of our proposed computational setup, as presented in Figure 3.

**Figure 3:**
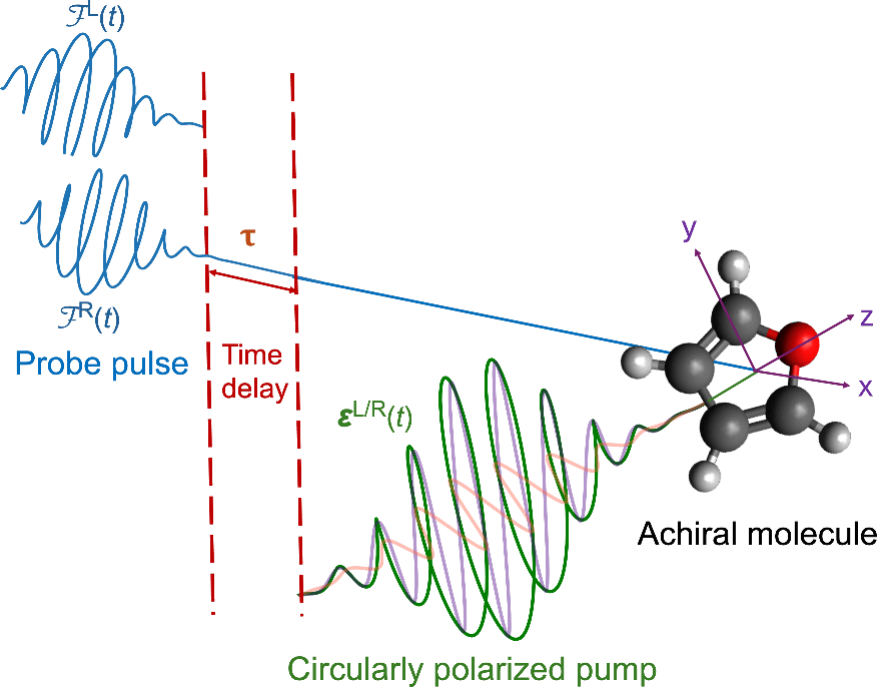
Schematic representation of the pump–probe setup for TR-ECD spectral simulation, where a probe pulse $F^{L/R}\left(t\right)$ is applied at a time delay of τ after the end of pump pulse $E^{L/R}\left(t\right)$ .

Theoretically, the ECD spectral function is calculated in the weak-probe regime from the imaginary part of the Rosenfeld tensor ***β*** (ω).^47–49,52,53^ In the real-time TDDFT framework, this tensor can be obtained by the Fourier transformation of the time-dependent induced magnetic dipole moment recorded during time propagation^54–56^

$$\beta_{kj}\left(\omega \right)=\frac{i}{\omega F_{0}\;f_{k}}\int_{-\infty }^{\infty }dtm_{j}^{\mathrm{ind}}\left(t\right)e^{i\omega t},,k,j\in x,y,z$$ 8

In this approach, the probe pulse is represented by an analytical Dirac delta function, $F\left(t\right)=F_{0}\;f\delta \left(t-t_{0}\right)$ , with amplitude $F_{0}$ , polarization vector ***f***, and initial time *t*_0_. We have extended this formalism to TR-ECD with the delta probe pulse applied at *t*_0_ = *T* + *τ*, where *T* is the pump pulse duration and *τ* is the time delay between the pump and probe pulses. To isolate the response of the chiral electronic wavepacket to the probe pulse only, we subtract the magnetic dipole moment induced by the pump pulse, in analogy with transient absorption spectroscopy.^57^ The essential theoretical background is discussed in detail in Supporting Information, Section S1.

The evolution of the chiral electronic wavepacket is monitored by varying the time delay between pump and probe pulses, as shown in Figure 4. TR-ECD spectra recorded immediately after the end of the pump pulse display mirror-image symmetry, as shown in Figure 4a, indicating an enantiomeric relationship between the CPL and CPR induced wavepackets. Figures 4b and 4c illustrate the full TR-ECD spectra as obtained with CPL (middle) and CPR (bottom) light, respectively. The spectra are rich in information, and the primary observations are as follows: (i) The mirror-image relationship between the CPL and CPR 2D signals is preserved over time. In other words, the helicity of the pump pulse induces an enantiomer-like relationship in the electronic wavepacket, and this relationship persists even during the rapid evolution of the chiral wavepacket. This observation is a direct consequence of the time dependence of the induced magnetic moment shown in Figure 2a and the mirror relationship between CPR and CPL driving. (ii) The system exhibits chirality flips (sign reversal of TR-ECD spectral lines) generally on attosecond timescales observed on horizontal cuts in Figures 4b and 4c. Notably, periods of these flips slightly vary for each energy window, likely connecting to various harmonics of the induced magnetic dipole moments presented in Figure 2. Furthermore, we also investigated TR-ECD spectra of other oriented molecular systems, namely, benzene and aniline. These results are relegated to Supporting Information, Section S6, and show similar chiral imprints induced by CPL and CPR pump pulses. These findings suggest that light-induced attosecond persistent electronic chirality in achiral systems is a robust and transferable phenomenon.

**Figure 4:**
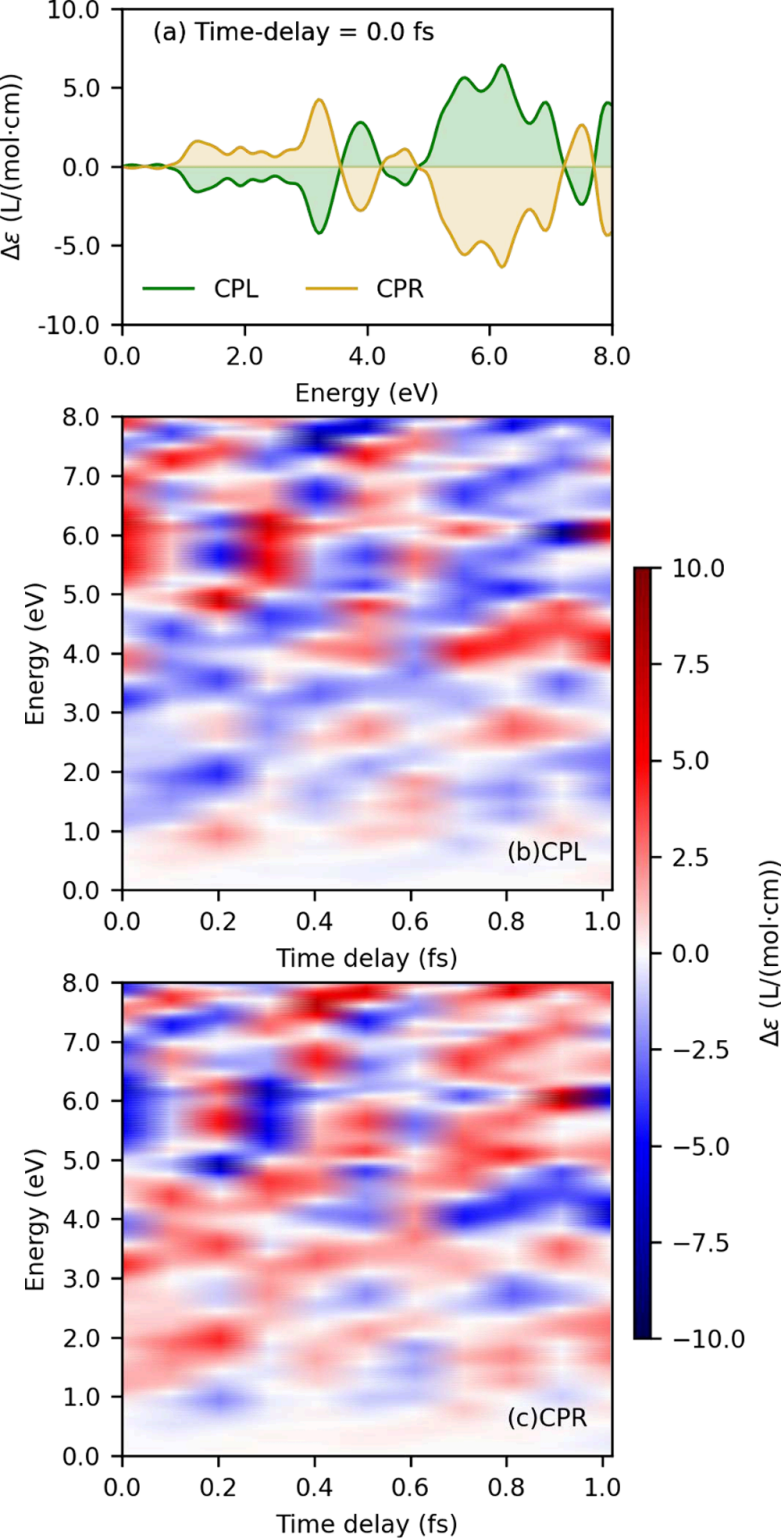
Time-resolved electronic circular dichroism (TR-ECD) spectra shown as differential extinction coefficient (Δ*ε*): (a) at zero pump–probe time delay (*τ* = 0) and (b, c) as a function of time delay *τ* for (b) circularly polarized left (CPL) and (c) circularly polarized right (CPR) pump pulses.

In summary, our work explored the ultrafast chiral dynamics of oriented achiral molecules driven by a chiral pump pulse, demonstrating how electronic currents and their induced magnetic dipole moments encode a time-resolved chiral signature and persistent chiral molecular states. The induced magnetic dipole moment shows nonvanishing oscillations even after the end of the pulse, maintaining an enantiomer-like symmetry between CPL- and CPR-induced electronic wavepackets. Therefore, flips of induced magnetic moments and the resulting magnetic fields may arise from flips of chiral (this work) and achiral^58^ electronic currents induced by circularly polarized laser pulses. An imprint of this oscillatory behavior of the current densities and magnetic dipole moments permits experimental observation by means of time-resolved electronic circular dichroism spectroscopy. We observe that the chiral response (both the magnetic dipole moment and the spectral function) does not oscillate with a characteristic frequency but rather emerges from a hierarchy of harmonic frequencies coherent with the pump laser. Unlike conventional HHG from the time-dependent electric dipole moment, the observed harmonics in the magnetic dipole moment stem from bound electronic currents, presenting an alternative route for probing and controlling chiral states, down to attosecond timescales. This method provides a spectral fingerprint for chiral wavepacket evolution, offering a powerful tool to study attosecond chiral dynamics in nonionized regimes and without changing achiral nuclear configuration. Looking ahead, we believe that our results will motivate future studies in connected attosecond charge migration,^59–62^ systems that are usually studied with photoemission and photodissociation, potentially linking to energy transfer mechanisms. The induced chiral transients could also be employed for a plethora of technological applications, from high selectivity ultrafast control of chemical reactions to light-induced phase transitions,^30^ electronics,^27^ and spintronics^28,29,63^

## Supplementary Material







## Notes

Methodology, computational setup, ground state depopulation, analysis of induced electric dipole moment, observations from the video showing the time evolution of induced charge and current density, and TR-ECD spectra of benzene and aniline (PDF)
A video showing the time evolution of the induced charge and current densities of furan (MOV)
Transparent Peer Review report available (PDF)
