# Supplementary material for: Light-Induced Persistent Electronic Chirality in Achiral Molecules Probed with Time-Resolved Electronic Circular Dichroism Spectroscopy
Source: J Phys Chem Lett. 2025 Aug 28;16(35):9210–6. doi: 10.1021/acs.jpclett.5c01808 (PMC12415891; doi:10.1021/acs.jpclett.5c01808)
Supplement: Supplementary file 1 [file jz5c01808_si_001.pdf]

Supporting Information: Light-Induced Persistent Electronic Chirality in Achiral Molecules Probed with Time-Resolved Electronic Circular Dichroism Spectroscopy

Torsha Moitra,[1,2] Lukas Konecny,[1,3,4] Marius Kadek,[1]
Ofer Neufeld,[5] Angel Rubio,[4,6] and Michal Repisky[2,1]

[1]*Hylleraas Centre for Quantum Molecular Sciences, Department of Chemistry, UiT The Arctic University of Norway, 9037 Tromsø, Norway*

[2]*Department of Physical and Theoretical Chemistry, Faculty of Natural Sciences, Comenius University, 84215 Bratislava, Slovakia*

[3]*Department of Inorganic Chemistry, Faculty of Natural Sciences, Comenius University, 84215 Bratislava, Slovakia*

[4]*Max Planck Institute for the Structure and Dynamics of Matter, Center for Free Electron Laser Science, Luruper Chaussee 149, 22761 Hamburg, Germany*

[5]*Technion Israel Institute of Technology, Faculty of Chemistry, Haifa 3200003, Israel*

[6]*Initiative for Computational Catalysis (ICC), The Flatiron Institute, 162 Fifth Avenue, New York, New York 10010, USA*

(Dated: August 1, 2025)

CONTENTS

S1. Methodology 3

S2. Computational setup 7

S3. Ground state depopulation 11

S4. Analysis of induced electric dipole moment 11

S5. Time evolution of induced charge and current density (video) 12

S6. TR-ECD spectra of benzene and aniline 13

References 14

S1. METHODOLOGY

Let us start the theory section by considering the interaction of a molecule with an external monochromatic radiation field characterized by the electric $\boldsymbol{E}(\boldsymbol{r},t)$ and magnetic $\boldsymbol{B}(\boldsymbol{r},t)$ field components. The radiation field induces oscillating electric and magnetic multipole moments in a molecule. These moments are related to components of the radiation field through molecular property tensors. A key quantity for predicting the optical rotatory dispersion (ORD) or the electronic circular dichroism (ECD) spectra is the property tensor $\boldsymbol{\beta}$, which relates the induced electric ($\boldsymbol{\mu}$) and magnetic ($\boldsymbol{m}$) dipole moments to the time derivative of the fields (in SI-based atomic units) [1, 2]:

$$\mu_j(t) = \int_{-\infty}^{\infty} dt'\ \alpha_{jk}(t-t')E_k(t') - \int_{-\infty}^{\infty} dt'\ \beta_{jk}(t-t')\frac{\partial B_k(t')}{\partial t'} + ... \tag{1}$$

$$m_j(t) = \int_{-\infty}^{\infty} dt'\ \chi_{jk}(t-t')B_k(t') + \int_{-\infty}^{\infty} dt'\ \beta_{kj}(t-t')\frac{\partial E_k(t')}{\partial t'} + ... \tag{2}$$

Here, $\boldsymbol{\alpha}$ and $\boldsymbol{\chi}$ are the electric polarizability and magnetic susceptibility tensors, respectively, and summations are implicit for repeated indices denoting the Cartesian axes (j,k). Note that the first index of the tensor $\boldsymbol{\beta}$ connects to an electric quantity, while the second index to a magnetic quantity.

The previous relations ignore the nonlocal response in space. This approximation is valid when the wavelength of the optical waves of interest is long compared to the range of the considered response function. This approximation is appropriate for the present work where the wavelength of visible light ≈ 5000 Å is much longer than the typical length scale of the organic molecules $\approx 10-100$ Å. Additional implication of this (long wavelength) approximation is that the electric field is spatially uniform and the magnetic field vanishes [2]. So, in the case of a polychromatic electric field given as

$$E_k(t) = \int_{-\infty}^{\infty} \frac{d\omega}{2\pi} E_k(\omega)e^{-i\omega t},$$

the property tensor $\boldsymbol{\beta}$ can be obtained within the approximation from Eq. (2)

$$\begin{aligned} m_j(t) &= \int_{-\infty}^{\infty} dt' \left[\int_{-\infty}^{\infty} \frac{d\omega}{2\pi}\beta_{kj}(\omega)e^{-i\omega(t-t')}\right]\left[-i\int_{-\infty}^{\infty}\frac{d\omega'}{2\pi}\omega' E_k(\omega')e^{-i\omega' t'}\right] \\ &= \frac{-i}{2\pi}\int_{-\infty}^{\infty} d\omega\ \beta_{kj}(\omega)\omega E_k(\omega)e^{-i\omega t}. \end{aligned} \tag{3}$$

Here, we used the identity $\int_{-\infty}^{\infty} dt\ e^{i(\omega-\omega')t} = 2\pi\delta(\omega-\omega')$. Now, as a particular case we consider an electric field with equal intensity (κ) for all frequencies and directions, *i.e.*, $E_k(\omega) = \kappa_k$. In the time domain this corresponds to the Dirac delta-type field $E_k(t) = \kappa_k\delta(t)$. By introducing the expression for $E_k(\omega)$ into the previous equation, and performing an inverse Fourier transform, we obtain

$$\begin{aligned}\int_{-\infty}^{\infty} dt\ m_j(t)e^{i\omega t} &= \frac{-i}{2\pi}\int_{-\infty}^{\infty} dt\ e^{i\omega t}\int_{-\infty}^{\infty} d\omega'\ \beta_{kj}(\omega')\omega' E_k(\omega')e^{-i\omega' t} \\ &= -i\omega\kappa_k\beta_{kj}(\omega). \end{aligned} \tag{4}$$

In other words, we see that the frequency-dependent property tensor $\boldsymbol{\beta}$ can be obtained from the time-dependent electrically induced magnetic dipole moment

$$\beta_{kj}(\omega) = \frac{i}{\omega\kappa_k}\int_{-\infty}^{\infty} dt\ m_j(t)e^{i\omega t}. \tag{5}$$

The final ECD spectra are obtained from the imaginary part of the isotropically averaged $\boldsymbol{\beta}$:

$$\bar{\beta}(\omega) = \frac{1}{3}\sum_k \beta_{kk}(\omega),$$

and reported in this work in terms of the differential extinction coefficient $\Delta\varepsilon$ in L/(mol·cm) [2, 3]:

$$\begin{aligned}\Delta\varepsilon(\omega) &= 10\frac{2N_A\omega^2}{\ln(10)c^2\epsilon_0}\operatorname{Im}\left[\bar{\beta}(\omega)\right] \\ &= 10\frac{2N_A}{\ln(10)c^2\epsilon_0}\omega\operatorname{Im}\left[\frac{i}{3}\sum_k\frac{m_k(\omega)}{\kappa_k}\right] \\ &= 10\frac{2N_A}{\ln(10)c^2\epsilon_0}\frac{e^2\cdot a_0}{m_\mathrm{e}}\omega^{\mathrm{AU}}\operatorname{Im}\left[\frac{i}{3}\sum_k\frac{m_k^{\mathrm{AU}}(\omega)}{\kappa_k^{\mathrm{AU}}}\right]. \end{aligned} \tag{6}$$

Here, N_A is the Avogadro constant, c is the speed of light in vacuum, ϵ_0 is the vacuum permittivity, a_0 is the Bohr radius, m_e is the electron mass, and e is the electron charge. All these physical quantities are expressed in SI units, except those with the superscript AU, which are expressed in SI-based atomic units (as obtained from our program's outputs).

The time-dependent induced magnetic dipole moment in Eq. (5) is calculated within the Kohn–Sham DFT framework as a trace of the magnetic dipole moment matrix $\mathbf{M}_j$ with the time-dependent density matrix $\mathbf{D}(t)$:

$$m_j(t) = \mathrm{Tr}\{\mathbf{M}_j\mathbf{D}(t)\} - m_j^{\mathrm{static}}. \tag{7}$$

Here, $m_j^{\rm static}$ refers to the static magnetic dipole moment obtained from the ground-state self-consistent field (SCF) density matrix $\mathbf{D}_0$ as: $m_j^{\rm static} = \mathrm{Tr}\{\mathbf{M}_j \mathbf{D}_0\}$. The matrix $\mathbf{M}_j$ consists of a finite-basis representation of the magnetic dipole moment operator ($\hat{\boldsymbol{m}}$), namely its orbital angular momentum part,

$$M_{j,ab} = -\frac{1}{2} \langle \chi_a | \left(\boldsymbol{r} \times \boldsymbol{p}\right)_j | \chi_b \rangle . \tag{8}$$

Since the present work restricts to closed-shell molecules, the spin angular momentum contribution to $\hat{\boldsymbol{m}}$ is negligible. The density matrix $\mathbf{D}(t)$ is obtained by solving the Liouville-von Neumann (LvN) equation of motion

$$i\frac{\partial \mathbf{D}(t)}{\partial t} = [\mathbf{F}(t), \mathbf{D}(t)], \tag{9}$$

where the time evolution of a system characterized by $\mathbf{D}(t)$ is driven by the Fock matrix $\mathbf{F}(t)$. The Fock matrix describes the molecular system itself as well its interaction with external field(s), and it consists of one-electron ($\mathbf{h}$), two-electron ($\mathbf{G}$), exchange–correlation ($\mathbf{V}^{\rm XC}$), and light–matter interaction ($\mathbf{V}^{\rm ext}$) contributions:

$$\mathbf{F}(t) = \mathbf{h} + \mathbf{G}[\mathbf{D}(t)] + \mathbf{V}^{\rm XC}[\mathbf{D}(t)] + \mathbf{V}^{\rm ext}(t). \tag{10}$$

All these matrices are represented in the basis of time-independent molecular orbitals (MOs) obtained from the solution of Kohn–Sham SCF equations, where each MO is composed of a linear combination of Gaussian-type orbitals (GTOs). In this work, we employed uncontracted aug-cc-pVTZ (for furan) and aug-cc-pVDZ (for aniline and benzene) GTOs [4, 5]. We employ the PBE0 exchange–correlation functional for furan, and the PBE functional for aniline and benzene [6, 7]. All implementation and numerical simulations pertaining to this work were performed using the ReSpect program [8].

As discussed above, molecular chiroptical effects can be attributed to the oscillating magnetic dipole moment induced by an external radiation electric field. The interaction of this external field with a molecular system is incorporated into our approach through the light–matter interaction term in Eq. (10):

$$\mathbf{V}^{\rm ext}(t) = -\mathbf{P}_k \mathcal{E}_k^{\rm L/R}(t) - \mathbf{P}_k \mathcal{F}_k(t). \tag{11}$$

In this work, we assume a time-resolved pump-probe experiment in the non-overlapping regime, where the system, characterized by the electric dipole moment ($\mathbf{P}$), interacts with both the

external electric pump ($\boldsymbol{\mathcal{E}}^{\mathrm{L/R}}$) and probe ($\boldsymbol{\mathcal{F}}$) fields. The probe pulse is applied at the end or after the pump pulse. The pump pulse has the form of a *chiral*, circularly polarized left (L) or right (R) Gaussian envelope function:

$$\boldsymbol{\mathcal{E}}^{\mathrm{L/R}}(t) = \begin{cases} \mathcal{E}_0 \boldsymbol{e}^{\mathrm{L/R}} \exp\left(\frac{-(t-t_0)^2}{2\sigma^2}\right) & t \leq T \\ 0 & t > T, \end{cases} \tag{12}$$

centered at t_0 and characterized by the amplitude $\mathcal{E}_0$, standard deviation σ, and helicity vector

$$\boldsymbol{e}^{\mathrm{L/R}} = \cos\big(\omega_0(t-t_0)\big)\boldsymbol{x} \mp \sin\big(\omega_0(t-t_0)\big)\boldsymbol{y}, \tag{13}$$

which oscillates with the carrier frequency ω_0. As the Gaussian envelope function decays asymptotically, we impose a step function for $\boldsymbol{\mathcal{E}}^{\mathrm{L/R}}(t)$ at $t > T$. The pump duration T corresponds to the time at which the pump amplitude reaches the value $10^{-2} \times \mathcal{E}_0$, and is related to the standard deviation σ as

$$T = 2\sigma \left\lfloor \sqrt{-2\ln(0.01)} \right\rfloor . \tag{14}$$

In accordance with the previous discussion, the probe pulse $\boldsymbol{\mathcal{F}}(t)$ is modeled as an analytical delta function with the amplitude $\mathcal{F}_0$, direction $\boldsymbol{f}$, and origin at $T + \tau$:

$$\boldsymbol{\mathcal{F}}(t) = \mathcal{F}_0 \boldsymbol{f} \delta\big(t - (T+\tau)\big), \tag{15}$$

where τ denotes the time delay between the pump and probe pulses. The specific values used in our simulations for the pump-probe pulse setup and real-time propagation are provided in Table S1. Finally, the electric dipole operator represented in a finite basis has the matrix form

$$P_{k,ab} = -\left\langle \chi_a \right| r_k \left| \chi_b \right\rangle , \tag{16}$$

where $\boldsymbol{r}$ is the electronic position operator.

In the pump-probe setup, one aims to compute the absorption of the probe pulse by a molecule that is initially irradiated by a pump, either simultaneously or with a given delay [9, 10]. For pump-probe ECD, the goal is therefore to determine the response of the magnetic dipole moment with ($\boldsymbol{m}^{\mathrm{pp}}$) and without ($\boldsymbol{m}^{\mathrm{p}}$) the probe pulse. The difference ($\Delta\boldsymbol{m}^{\text{TR-ECD}}$) represents the excess of magnetization, which is responsible for the absorption of the probe. Using Eqs. (7) and (8), this requires evaluating

$$\Delta m_j^{\text{TR-ECD}}(t) = m_j^{\mathrm{pp}}(t) - m_j^{\mathrm{p}}(t) = \mathrm{Tr}\big\{\mathbf{M}_j\big(\mathbf{D}^{\mathrm{pp}}(t) - \mathbf{D}^{\mathrm{p}}(t)\big)\big\} , \tag{17}$$

where $\mathbf{D}^{\mathrm{p}}(t)$ and $\mathbf{D}^{\mathrm{pp}}(t)$ are time-dependent density matrices obtained from two distinct simulations: one with only the pump pulse (p) and one with both the pump and probe pulses (pp). Finally, the vector $\Delta\boldsymbol{m}^{\text{TR-ECD}}(t)$ is transformed to the frequency domain using the discrete Fourier transformation

$$\Delta\boldsymbol{m}^{\text{TR-ECD}}(\omega_a) = \sum_{b=0}^{N-1} \Delta t \Delta\boldsymbol{m}^{\text{TR-ECD}}(t_b) \exp(-\gamma t_b) \exp\left(i2\pi\frac{ab}{N}\right). \tag{18}$$

In the above equation, γ is the real-valued damping constant and the frequency index a runs from 0 to $(N-1)$ where N is the number of time-steps in the real-time simulation and $\omega_a = \frac{2\pi a}{\Delta t}$. Note here, that for the Fourier transformation $t = 0$ is set to the time of application of probe pulse, *i.e.*, $T + \tau$.

The final TR-ECD spectra were obtained from three independent pump-probe simulations with the uni-directional pump field and probe field oriented along three Cartesian directions. This allows us to construct full frequency-dependent property tensor $\boldsymbol{\beta}$ in the sense of Eq. (5). Its isotropically averaged imaginary part leads to the TR-ECD differential extinction coefficient as in Eq. (6).

S2. COMPUTATIONAL SETUP

All real-time TDDFT simulations employed the theoretical methodology discussed in the previous section, and were performed with 0.2 au time-step (Δt) for 20000 steps, resulting in a total simulation time of 96.75 fs. The UV-vis spectra is obtained with a δ-type pulse of amplitude 0.001 au. The details of the pump and probe pulse parameters for the time-resolved studies are summarized in Table S1. The experimental geometry was used for the furan, aniline and benzene molecules [11], as given in Table S2.

In addition, both the time-domain and Fourier-transformed frequency-domain representations of the broadband circularly polarized left- and right-handed pump pulses are shown in Figs. S1-S3. The carrier frequency (ω_0) of the pump pulse is tuned to the first excited state (unless otherwise mentioned), while the pulse duration is chosen so that its frequency bandwidth also encompasses the second bright excited state. This results in more intricate coherent dynamics within the molecule.

Table S1. Summary of pump and probe pulse parameters used in all simulations. Mathematical forms of these pulses are given in Eqs. (12)-(15), and lists as: carrier frequency (ω_0), pump amplitude ($\mathcal{E}_0$), pump center (t_0), pump duration (T), standard deviation (σ), and probe amplitude $\mathcal{F}_0$. The pulse shapes are presented in Figures S1-S3.

Molecule	$\boldsymbol{\mathcal{E}}^{\mathrm{L/R}}(t)$										$\boldsymbol{\mathcal{F}}(t)$	
	ω_0		$\mathcal{E}_0$		t_0		T		σ		$\mathcal{F}_0$	
	au	eV	au	V/m	au	fs	au	fs	au	fs	au	V/m
Furan (C_4H_4O)	0.223	6.07	0.03	1.54×10^{10} [a]	84.6	2.05	169.2	4.09	28.2	0.68	0.001	5.14×10^8 [b]
Aniline ($C_6H_5NH_2$)	0.236[c]	6.41	0.03	1.54×10^{10}[a]	79.8	1.93	159.6	3.86	26.60	0.64	0.001	5.14×10^8 [b]
Benzene (C_6H_6)	0.251	6.84	0.03	1.54×10^{10}[a]	75.0	1.81	150.0	3.63	25.0	0.60	0.001	5.14×10^8 [b]

[a] $I_0 = 3.16 \times 10^{13}$ W/cm^2

[b] $I_0 = 3.51 \times 10^{10}$ W/cm^2

[c] Chosen as it is has the maximum intensity and is similar in energy with C_6H_6.

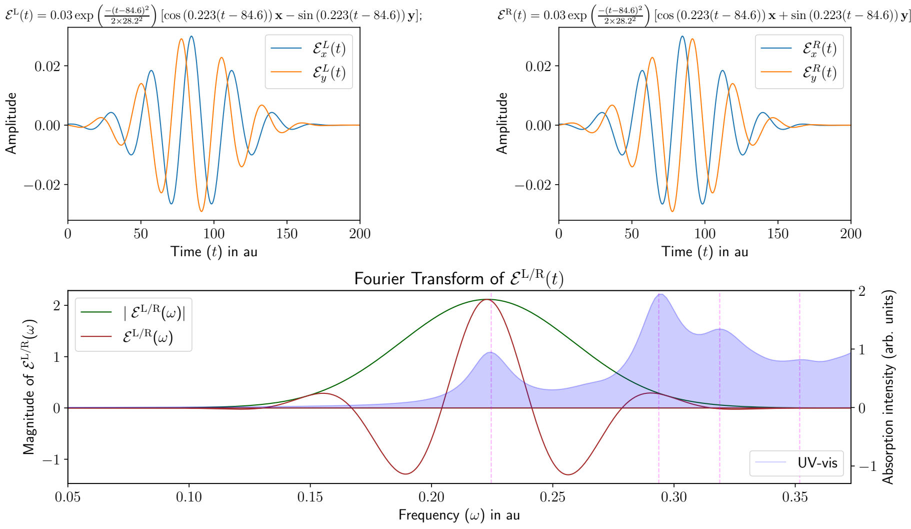

Figure S1. Furan: CPL (top left) and CPR (top right) pump features in time (top) and frequency (bottom) domain. The ground state bright excitation energies are marked by magenta dashed lines.

Table S2. Cartesian coordinates of the molecules (in Å) [11]

Furan				Benzene				Aniline			
	X	Y	Z		X	Y	Z		X	Y	Z
O	0.0000	0.0000	1.1626	H	0.0000	2.4750	0.0000	C	-0.2201	-1.1999	-0.0049
C	0.0000	1.0920	0.3487	C	0.0000	1.3935	0.0000	C	1.1643	-1.1947	0.0035
C	0.0000	-1.0920	0.3487	C	0.0000	0.6967	1.2069	C	1.8704	0.0000	0.0075
C	0.0000	0.7169	-0.9596	H	0.0000	1.2375	2.1434	H	1.6966	-2.1387	0.0075
C	0.0000	-0.7169	-0.9596	C	0.0000	-0.6967	1.2069	C	1.1643	1.1947	0.0035
H	0.0000	2.0473	0.8439	H	0.0000	-1.2375	2.1434	H	2.9528	0.0000	0.0143
H	0.0000	-2.0473	0.8439	C	0.0000	-1.3936	0.0000	C	-0.2201	1.1999	-0.0049
H	0.0000	1.3509	-1.8290	H	0.0000	-2.4750	0.0000	H	1.6966	2.1387	0.0075
H	0.0000	-1.3509	-1.8290	C	0.0000	-0.6968	-1.2069	C	-0.9339	-0.0000	-0.0084
				H	0.0000	-1.2375	-2.1434	H	-0.7591	2.1414	-0.0130
				C	0.0000	0.6968	-1.2069	H	-0.7591	-2.1414	-0.0130
				H	0.0000	1.2375	-2.1434	N	-2.3199	0.0000	-0.0724
								H	-2.7690	0.8355	0.2628
								H	-2.7690	-0.8355	0.2628

Molecule	Level	IE $= -\epsilon^{\mathrm{HOMO}}$
Furan	PBE0/aug-cc-pVTZ	0.24559 au
Aniline	PBE/aug-cc-pVDZ	0.18236 au
Benzene	PBE/aug-cc-pVDZ	0.23240 au

Table S3. Ionization energies from Koopman´s theorem calculated as the negative of the highest occupied KS orbital energy. Although more rigorous methodologies, like Δ-SCF, are available, we do not pursue them as the energies are only a qualitative energy boundary and are not used for any analysis.

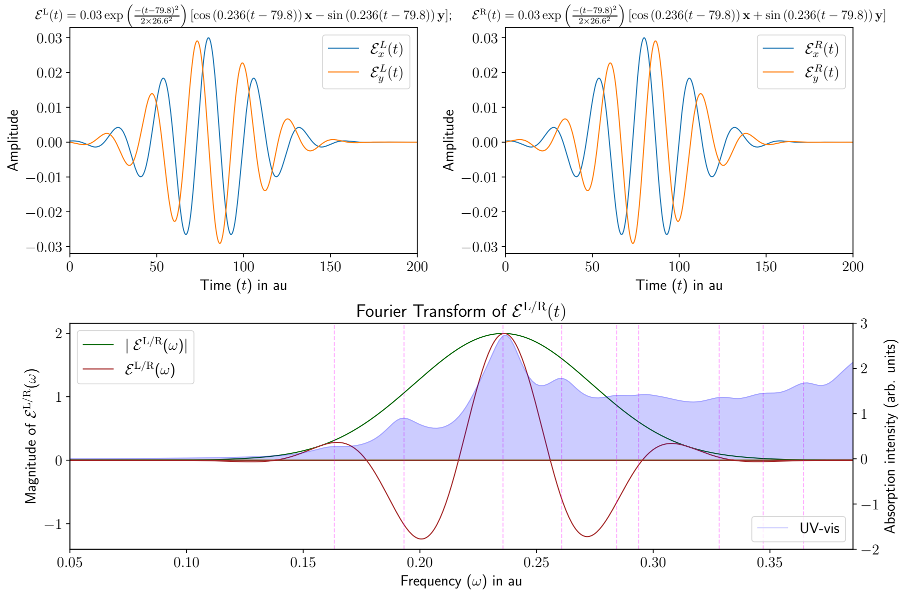

Figure S2. Aniline: CPL (top left) and CPR (top right) pump features in time (top) and frequency (bottom) domain. The ground state bright excitation energies are marked by magenta dashed lines.

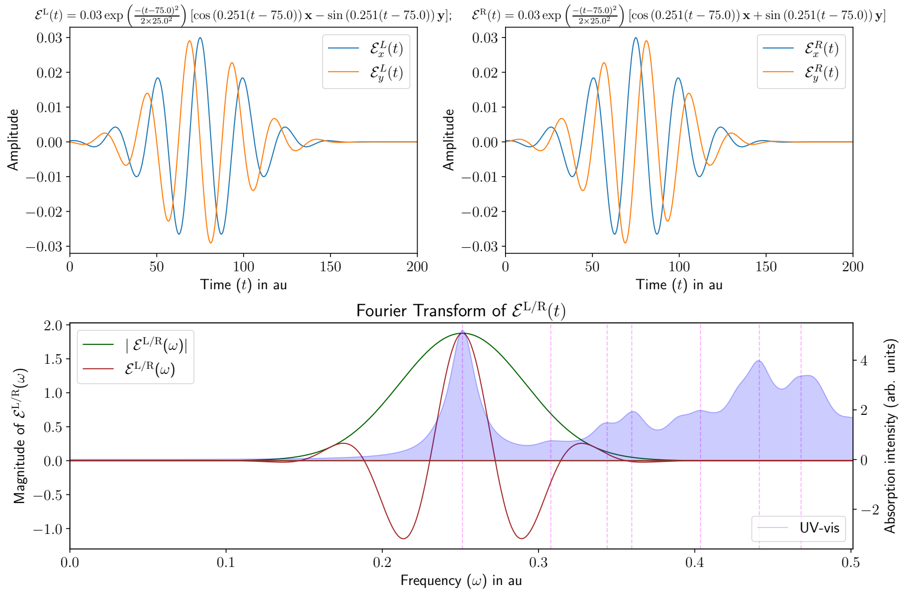

Figure S3. Benzene: CPL (top left) and CPR (top right) pump features in time (top) and frequency (bottom) domain. The ground state bright excitation energies are marked by magenta dashed lines.

S3. GROUND STATE DEPOPULATION

We obtain the fractional ground state population as $\mathrm{Tr}[\mathbf{D}_0\mathbf{D}(t)]$. For exact theory, the ground-

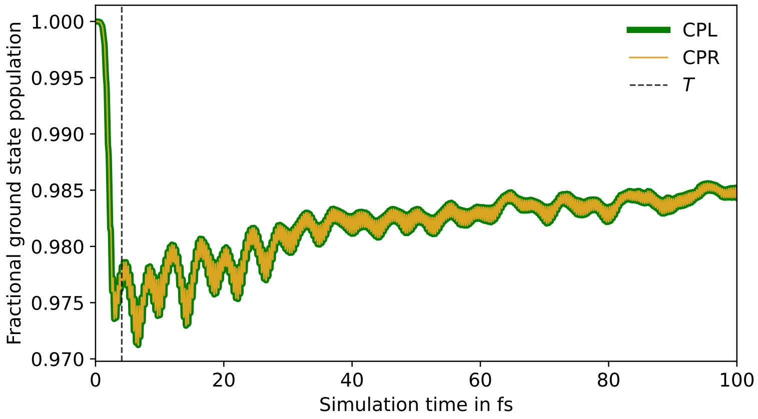

Figure S4. Furan: Variation in the fractional contribution of ground state towards the chiral electronic wavepacket computed as $\mathrm{Tr}[\mathbf{D}_0\mathbf{D}(t)]$ with simulation time (t). $\mathbf{D}_0$ and $\mathbf{D}(t)$ are the reduced one-electron density of the ground state at time zero and non-stationary state at time t, respectively.

state population after the end of pump and probe pulses should be constant. However, this does not hold for approximate mean-field methods such as HF or DFT, resulting in artificial oscillations in ground-state population, as seen in Fig. S4 and discussed in further details in Ref. [10, 12, 13].

As is well established in transient absorption spectroscopy, sufficient ground-state depopulation is crucial for generating an electronic wavepacket with distinct characteristics and a unique spectral signature [10]. Fig. S4 shows that the pump pulse setup of our choice leads to a ground-state depopulation of approximately 3%. Following the pump pulse, the electronic wavepacket undergoes coherent dephasing, giving the appearance of relaxation toward the ground-state density due to destructive interference among its eigenstate components.

S4. ANALYSIS OF INDUCED ELECTRIC DIPOLE MOMENT

As discussed in the Letter, the oscillations in the induced magnetic moment (shown in Fig. 2) is not characterized by a single dominant frequency, instead it comprises of multiple harmonic

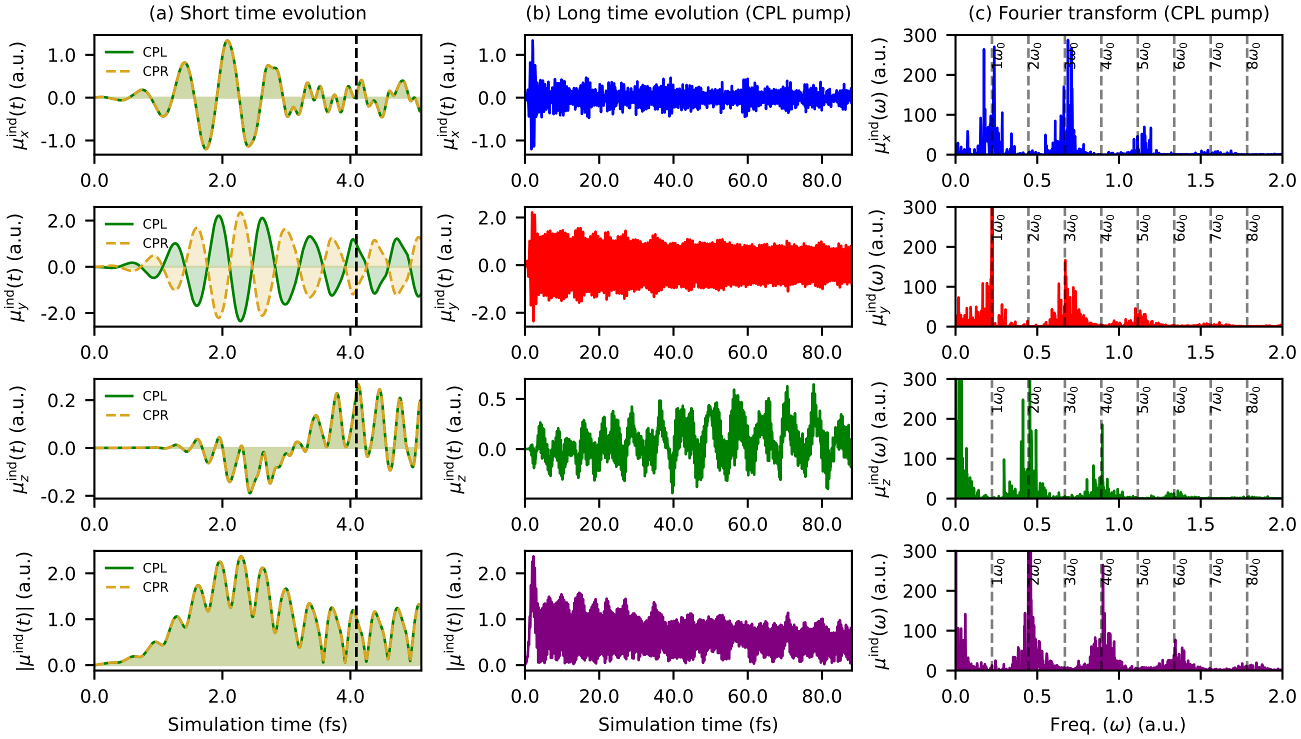

Figure S5. Furan: Short time evolution (a) and long time evolution (b) of the magnitude ($|\boldsymbol{\mu}^{\rm ind}(t)|$) and components of electric dipole moment $\mu^{\rm ind}(t) = (\mu_x^{\rm ind}(t), \mu_y^{\rm ind}(t), \mu_z^{\rm ind}(t))$ induced by CPL and/or CPR pump pulses. The black dashed line in (a) at 4.09 fs marks the end of the pump pulse, after which the electronic wavepacket evolves freely. (c) Fourier transform of the induced electric moment obtained from the long time evolution in (b). The harmonic orders of the carrier frequency $\omega_0 = 0.223$ au are marked by dashed lines.

orders of the carrier frequency (ω_0). A similar behavior is observed for the induced electric dipole moment $\mu(t)$ signal as shown in Figure S5, as is more commonly known for high harmonic generation (HHG) [14–16].

S5. TIME EVOLUTION OF INDUCED CHARGE AND CURRENT DENSITY (VIDEO)

A video showing the time evolution of the induced charge and current densities of furan are attached (Fig. SI_charge_current.mov). The primary observations are: (i) The enantiomer-like relationship between the electronic wavepackets induced by CPL and CPR is maintained both during and after the pump pulse. (ii) Notably, the induced chiral current density persists even

after the pump pulse has ended, allowing its detection.

S6. TR-ECD SPECTRA OF BENZENE AND ANILINE

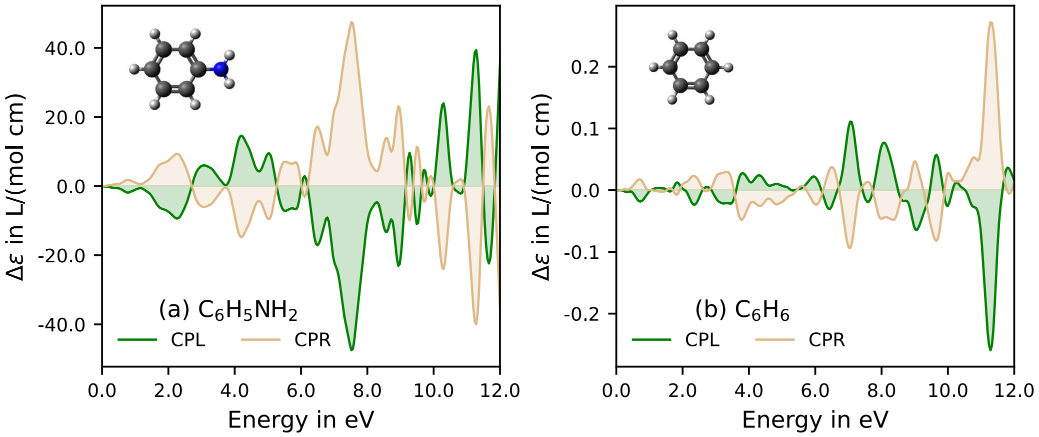

Figure S6. Time-resolved electronic circular dichroism spectrum as differential extinction coefficient ($\Delta\varepsilon$) of (a) aniline ($C_6H_5NH_2$) and (b) benzene (C_6H_6) obtained at time-delay $\tau = 0.0$. The pump-probe setup parameters are described in Table S1.

We generalize the proposed technique by further investigating planar achiral benzene and aniline molecules. Note that the relative orientation of the pump and molecule differs between the furan/benzene case (light propagates along z within the molecular plane) and the aniline case (light propagates along z perpendicular to the molecular plane). The TR-ECD spectra obtained for the molecules at time-delay $\tau = 0.0$ fs is shown in Fig. S6. We observe a mirror-image relationship between the CPL and CPR induced spectral function, concurrent with observations for furan molecule. However, our study on furan along with literature reports on NaK [17], suggests a hypothesis that a ground state static electric dipole moment may be necessary for observing induced electronic chirality. Our simulations on benzene and aniline reveal that while chirality is indeed induced in achiral benzene using a monochromatic CP light pump pulse, the

spectral intensity is two orders of magnitude smaller than that of aniline.

---

[1] D. Varsano, L. A. Espinosa-Leal, X. Andrade, M. A. Marques, R. Di Felice, and A. Rubio, Phys. Chem. Chem. Phys. **11**, 4481 (2009).

[2] L. D. Barron, *Molecular light scattering and optical activity* (Cambridge University Press, 2009).

[3] L. Konecny, M. Repisky, K. Ruud, and S. Komorovsky, J. Chem. Phys. **151**, 194112 (2019).

[4] T. H. Dunning Jr, J. Chem. Phys. **90**, 1007 (1989).

[5] R. A. Kendall, T. H. Dunning, and R. J. Harrison, J. Chem. Phys. **96**, 6796 (1992).

[6] C. Adamo and V. Barone, J. Chem. Phys. **110**, 6158 (1999).

[7] J. P. Perdew, K. Burke, and M. Ernzerhof, Phys. Rev. Lett. **77**, 3865 (1996).

[8] M. Repisky, S. Komorovsky, M. Kadek, L. Konecny, U. Ekström, E. Malkin, M. Kaupp, K. Ruud, O. L. Malkina, and V. G. Malkin, J. Chem. Phys. **152**, 184101 (2020).

[9] U. De Giovannini, G. Brunetto, A. Castro, J. Walkenhorst, and A. Rubio, ChemPhysChem. **14**, 1363 (2013).

[10] T. Moitra, L. Konecny, M. Kadek, A. Rubio, and M. Repisky, J. Phys. Chem. Lett. **14**, 1714 (2023).

[11] National institute of standards and technology (NIST) experimental geometry data, accessed: January, 2024.

[12] J. I. Fuks, N. Helbig, I. Tokatly, and A. Rubio, Phys. Rev. B **84**, 075107 (2011).

[13] J. I. Fuks, K. Luo, E. D. Sandoval, and N. T. Maitra, Phys. Rev. Lett. **114**, 183002 (2015).

[14] O. Neufeld, D. Ayuso, P. Decleva, M. Y. Ivanov, O. Smirnova, and O. Cohen, Phys. Rev. X. **9**, 031002 (2019).

[15] D. Ayuso, A. F. Ordonez, P. Decleva, M. Ivanov, and O. Smirnova, Opt. Express **30**, 4659 (2022).

[16] R. Cireasa, A. Boguslavskiy, B. Pons, M. Wong, D. Descamps, S. Petit, H. Ruf, N. Thiré, A. Ferré, J. Suarez, *et al.*, Nat. Phys. **11**, 654 (2015).

[17] Y. Chen, D. Haase, J. Manz, H. Wang, and Y. Yang, Nat. Comm. **15**, 565 (2024).
