# Supplementary material for: Light-Induced Persistent Electronic Chirality in Achiral Molecules Probed with Time-Resolved Electronic Circular Dichroism Spectroscopy
Source: J Phys Chem Lett. 2025 Aug 28;16(35):9210–6. doi: 10.1021/acs.jpclett.5c01808 (PMC12415891; doi:10.1021/acs.jpclett.5c01808)
Supplement: Supplementary file 3 [file jz5c01808_si_004.pdf]

jz-2025-01808t.R1

Name: Peer Review Information for "Light-Induced Persistent Electronic Chirality in Achiral Molecules Probed with Time-Resolved Electronic Circular Dichroism Spectroscopy"

First Round of Reviewer Comments

Reviewer: 1

Comments to the Author

The idea of inducing (electronic) chirality by circularly polarized light that is presented in this paper is original and interesting. The simulation techniques should be adequate to demonstrate the effect, my main comments relate to the idealized model that is used compared to what will be possible experimentally. I believe it would be of interest to discuss issues related to realising an experimental set up, that can approach this theoretical model before making the jump to future technological applications. Also I wonder about the effect of molecular motion that is neglected in the current model. In particular:

1) The calculations assume perfect alignment of the molecules prior to absorption of the pulse. As this will be hard to realise in practice, it would be instructive to also include some calculations of molecules that are not perfectly aligned and see how averaging over such configurations would affect the observable signal.

2) Molecular vibrations are likely to exert an effect as the simulation time is sufficiently long to cover a few periods of vibrations. This will have an impact on the electron dynamics, and also adds an additional source of randomness as the nuclear positions (viewed in a classical Born-Oppenheimer picture) will not always display the perfect achiral symmetry of the optimized structure, but some random sampling around that minimum point on the Born-Oppenheimer surface.

3) The calculations appear to be noiseless as Figure 4 shows exact mirror images. This gives faith in the numerical precision of the employed algorithm, but I wonder what deliberately adding some random noise (to simulate weak intermolecular interactions) could give. At what level of noise does the mirror symmetry disappear ? This may tell something about the robustness of the effect and the chance to observe this in finite temperature experiments.

Reviewer: 2

Comments to the Author

Referee report on the manuscript

"Light-Induced Persistent Electronic Chirality in Achiral Molecules Probed with Transient Absorption Circular Dichroism Spectroscopy"

submitted for publication in the Journal of Physical Chemistry Letters
(manuscript ID = jz-2025-01808t) by T. Moitra, L. Konecny, M. Kadek, O. Neufeld, A. Rubio

and M. Repisky

General: The authors use quantum chemistry and quantum dynamics to simulate the excitation of a small fraction (≈ 3%) of an oriented achiral molecule from the ground state to excited states by means of a single ultrashort (few fs) right or left polarized vis/UV laser pump pulse, cf. Figure 1(a) and Figure S4 and Table S1 of Supporting Information (SI). The ultrashort duration corresponds to a broad spectral width (> 1 eV) such that the laser excites at least two excited states, cf. Figures S1, S2, S3 of SI. At the end of the pulse, the molecule is thus prepared in a non-stationary superposition of at least three electronic states. The helicity of the laser pulse is transferred to corresponding electronic ring currents, cf. Figure 1(c). The related electronic densities are chiral, cf. Figure 1(b), with quasiperiodic chirality flips in the time domain of the period of the carrier frequency of the laser pulse (typically 1 fs). The electronic ring currents induce magnetic dipole moments which flip synchronously, cf. Figure 2. The authors suggest monitoring these magnetic dipole moments by means of an ultrafast (delta-type) probe pulse, cf. Figures 3 and 4, that means by time-resolved electronic dichroisms (TR-ECD), also called transient absorption dichroism spectroscopy. The authors consider the molecular model with the nuclei frozen in the experimental geometry of the electronic ground state, cf. Table S2 of SI. The resulting

ring currents and the laser induced flips of the magnetic dipole moments persist for at least 100 fs, cf. Figure 2(a),(b). The corresponding Fourier transforms yields patterns which remind of high harmonic generation, cf. Figure 2(c). In SI, the authors also calculate corresponding time evolutions of the electric dipole moments, yielding similar time evolutions as well as high harmonic type patterns, cf. Figure S5 of SI. The underlying theory is outlined in Section S1 of the Supporting Information (SI). Applications are for oriented achiral furan (main text) as well as for oriented benzene and aniline (SI).

The field of research on laser induced chirality flips in molecules is a rather young hot topic, cf. Refs. (8),(9). The authors add important progress to this rapidly evolving field: (i) excitation of at least two electronic states by a single ultrashort circularly polarized laser pulse (ii) evaluation of the laser-induced ring currents and induced magnetic moments (iii) monitoring signatures of the chirality flips by means of ultrafast pump probe spectroscopy, i. e. TR-ECD. On the way, they discover (iv) high harmonic type spectra of the induced magnetic dipoles as well as the induced electric dipoles. (v) The results are achieved by means of impressive advances of the fundamental theory and using the ReSpect program which have been developed by A. Rubio and his partners since 2009, cf. Refs. [1], [3], [6] – [8], [10],[11] of SI.

The resume is that this is excellent work which adds enormous progress of the field of electronic chirality flips in achiral molecules. The authors are to be congratulated, and the J. Phys. Chem. Letters should be proud of publishing this fascinating work. Before publication, the authors should consider, however, the following list of fourteen

Special items.

1) Title and TOC Graphic: Please use consistent terminology. Interested non-experts would understand the acronym “TR-ECD” in the TOC Graphic more easily if in the title, “transient absorption circular dichroism spectroscopy” is replaced by “time resolved electronic circular dichroism spectroscopy”, in accord with e.g. the Figure legends 4, S6.
2) TOC Graphic and Figure 1(c) and Video: There are myriads of arrows which should illustrate the flux densities of the ring currents, but these arrows are exceedingly small so that it is impossible for me
(and presumably for many readers) to extract the patterns of the

   fluxes. The authors should replace this worthless presentation of the flux densities with a clear one.

3) The authors should remind the reader, not only in the main text but also in the Abstract and in the Conclusions, that they consider the scenario of molecules with frozen nuclei. In contrast, vibrations in the electronic ground and excited states may deplete the overlap of the representative vibrational wave functions, and this would cause decoherence of the phenomena which are discovered by the authors. These effects are discussed for electronic chirality flips in RbCs, cf. G. Liu et al, ChemPhysChem 2024, 25, e202400595.

4) The authors should point to the following caveat, in the Conclusion Section: Flips of induced magnetic moments and the resulting magnetic fields may arise from flips of chiral (this work) as well as from achiral electronic currents induced by circularly polarized laser pulses, see the discussion in I. Barth and J. Manz, "Quantum Switching of Magnetic Fields by Circularly Polarized Re-Optimized pi Laser Pulses: From One-Electron Atomic Ions to Molecules", in

"Progress in Ultrafast Intense Laser Science VI", Springer Series in Chemical Physics, Vol 99, 21-44, eds K. Yamanouchi, A. D. Bandrauk and G. Gerber (Springer, Berlin, 2010). Turning the table, the results presented by the authors may be considered as signatures, but not necessarily as proof of "light-induced persistent electronic chirality in achiral molecules".

5) The authors should comment on the following discrepancy of the effects of single circularly polarized laser pulses. On the one hand, they show that these laser pulses may induce alternating ring currents which in turn induce flipping magnetic moments. On the other hand, these laser pulses may also induce uni-directional electronic ring currents without any flips, see e.g. Ref. (11) together with I. Barth and J. Manz, Angew. Chem. Int. Ed. 2006, 45, 2962 and G. Hermann et al, J. Phys. Chem. A 2016, 120, 5360. The carrier frequencies of the laser pulses for these "non-flipping" scenarios are resonant to excitations of selected doubly degenerate states, e.g. in benzene (... which is also considered by the authors, but with different scenario of the laser pulses, see below). In contrast, Fujimura and coworkers investigated excitations of two nondegenerate states in aromatic molecules by means of ultrashort linearly polarized laser pulses, and they discovered flips of the electronic ring currents, see e.g. H. Mineo, S.H. Lin , Y. Fujimura, Chem Phys. 2014, 422, 103 and earlier work cited therein. This discrepancy suggests a working hypothesis, namely a necessary (but not sufficient) condition for inducing flips of the ring currents and the induced magnetic fields is simultaneous excitations of at least two non-degenerate states. Gratifyingly, this condition is satisfied by the authors' application of ultrashort laser pulses (≈ 4 fs) with broad spectral width (> 1 eV).

6) Along with Refs. (3-7), the authors should quote pioneering work by Koch, Leibscher and coworkers, e.g. D. S. Tikhonov et al, Pump-probe spectroscopy of chiral vibrational dynamics, Sci. Adv. 2022, 8, eade0311 and M. Leibscher et al, Quantum control of rovibrational dynamics of chiral vibrational dynamics, PRA 2024, 109, 012810.

7) The authors should clarify the following discrepancy: In Section S1 of SI, they consider "an electric field with equal intensity for all frequencies and directions". The derivations rest on the validity of this assumption. Accordingly, "the final TR-ECD spectra were obtained from three independent pump-probe simulations with the probe field oriented along three Cartesian directions." But the pump-field is unidirectional!

8) Can equation (14) in SI be simplified? (It contains the expression $\sqrt{sigma^2}/sigma$).

9) In Table S1 of SI, the authors should specify the maximum intensity of the pump and probe laser pulses (e.g. in a footnote, in units of W/cm^2). The value for the pump pulse is given in the main text, but it will be helpful to list it also in Table S1 of SI.

10) Table S2 of SI has the Cartesian coordinates for furan in the YZplane, in accordance with Figure 1(a). But for benzene and aniline, it is in the XY plane or almost in the XY plane. If the pump laser pulse propagates along Z, as illustrated in Figure 1(a), then the scenario for benzene and for analine is entirely different from furan. In fact, the scenario for benzene would be analogous to the scenario of G. Hermann et al, J. Phys. Chem. A 2016, 120, 5360, and one would expect similar patterns of the ring currents, not entirely different ones. The authors should check the Cartesian coordinates in Table S2- perhaps they need to be permutated from XYZ to YZX?

11) Figures S1, S2, S3 of SI compare the frequency domain of the circularly polarized laser pulses with the molecular absorption spectra and the bright excitation energies. The authors should explain how they calculated these absorption spectra and excitation energies. Else they should quote the original publications of these spectra and energies.

12) The chiral molecular-current analogue to high harmonic generation HHG which is illustrated in Figures 2(c) and S5(c) extends to rather high energies. For comparison, the authors should also list the ionization potentials IP. If the energies of the HHG type spectra exceed IP, then the authors should comment on the possibility that their laser pulses might destroy their molecules by ionization.

13) Section S5 of SI points to two primary observations (i) and (ii) of the video. Please add another set of snapshots for slow-motion camera of one cycle of the chirality switch of the electronic densities and flux densities, after the end of the laser pulse.

14) The last sentence of Section S3 of SI mentions “wavepacket ... relaxation due to decoherence, gradually evolving toward the groundstate electronic density.” The authors should specify the mechanism of decoherence.

Reviewer: 3

Comments to the Author

This is a fascinating and well-written manuscript on a potentially new spectroscopic phenomenon, namely the generation of persistent chiral electronic currents in oriented achiral molecules, which may provide a new route to controlled attosecond chiral dynamics. Through real-time TD-DFT simulations of furan (with benzene and aniline reported in the SI), the authors examine oscillations in the magnietic dipole moment and observe a set of absorptions at the harmonics of the carrier frequency of the external circularly polarized field.

The manuscript is clearly written with sufficient information (including the SI) for others to reproduce the work. The results are compelling, and the analysis is insightful. I strongly recommend publication in JPCL after the authors have addressed the following minor points:

(1) Eq. (1): Please explicitly tie the L/R designation to -/+ in the field.

(2) p.8: "Contrarily, the chiral-current harmonics do not cause light emission, but tune the speed with which the molecular handedness can ultimately be controlled." What is the evidence that the harmonics do not cause light emission? What part of the simulation includes emission dynamics upon which this statement would be supported?

(3) p.11: "delegated" --> "relegated"

(4) p.12: "this microscopic behaviors" --> "this microscopic behavior"

Author's Response to Peer Review Comments:

Senior Editor
The Journal of Physical Chemistry Letters

Date: 16/07/2025

Dear Editor,

We would like to express our sincere gratitude to the reviewers for their thorough evaluation of our work and for providing numerous insightful and constructive comments. We also appreciate the reviewers' recognition of the theoretical and computational advancements presented in the manuscript, as well as their identification of its key contributions. In the following, we provide detailed responses to each comment raised by the reviewers and the editorial office. For clarity, each comment is first restated in full, followed by our corresponding response. Additionally, marked-up versions of the manuscript and Supporting Information, highlighting all revisions relative to the original submission, have been prepared and are provided in a separate file. Additionally, we are pleased to submit a cover art and kindly request its consideration for the journal's front cover.

Yours sincerely,
Michal Repisky

Reviewer 1:

Report: The idea of inducing (electronic) chirality by circularly polarized light that is presented in this paper is original and interesting. The simulation techniques should be adequate to demonstrate the effect, my main comments relate to the idealized model that is used compared to what will be possible experimentally. I believe it would be of interest to discuss issues related to realising an experimental set up, that can approach this theoretical model before making the jump to future technological applications. Also I wonder about the effect of molecular motion that is neglected in the current model. In particular:

Question 1: The calculations assume perfect alignment of the molecules prior to absorption of the pulse. As this will be hard to realise in practice, it would be instructive to also include some calculations of molecules that are not perfectly aligned and see how averaging over such configurations would affect the observable signal.

Response: Indeed, the present computational experiment assumes an isolated molecular system with a controlled alignment relative to the initial pump pulse (see graphical sketch in Figure 1a of the manuscript). We agree with the reviewer that these ideal experimental conditions are currently challenging, though not impossible to achieve in practice (S. Fleischer, *et al.*, *Phys. Rev. Lett.* 107, 163603, **2011**). However, the primary goal of the manuscript is to elucidate and demonstrate the fundamental physical processes occurring in achiral molecular systems irradiated by chiral light. Furthermore, we propose an experimental setup that could enable the observation of these processes, taking advantage of ongoing advances in experimental techniques.

To address both our own and the reviewer's interest, we carried out additional simulations in which the molecular system was systematically rotated relative to the pump pulse. Preliminary results indicate that the chiral signals remain robust and that the time-resolved ECD spectra exhibit more intricate enantiomeric relationships. A comprehensive and quantitative assessment of orientation effects, however, would necessitate a further investigation, which lies beyond the scope of the present work. Ongoing efforts in our laboratory are directed toward elucidating these effects, and we intend to report the findings in a separate publication.

Question 2: Molecular vibrations are likely to exert an effect as the simulation time is sufficiently long to cover a few periods of vibrations. This will have an impact on the electron dynamics, and also adds an additional source of randomness as the nuclear positions (viewed in a classical Born-Oppenheimer picture) will not always display the perfect achiral symmetry of the optimized structure, but some random sampling around that minimum point on the Born-Oppenheimer surface.

Response: The time-delay between the pump and probe pulses within which the effect of light-induced chirality is reported in this Letter is approximately 1 fs. In furan, the highest-frequency vibrational modes correspond to C–H stretching vibrations, with frequencies around 3150 cm^{-1} (Billes *et al.*, *J. Mol. Struct. THEOCHEM* 672, 1, **2004**). This translates to a vibrational period of roughly 10 fs. Other modes, such as ring puckering or C=C stretching,

exhibit even longer characteristic timescales in the range of 20–50 fs. This vibrational timescale is further supported by ultrafast nuclear dynamics studies on furan by Fuji *et al.*, *J. Chem. Phys.* 133, 234303, **2010**. As a result, deviations from the optimized achiral geometry due to nuclear motion are expected to be negligible within this timescale. However, we acknowledge that over longer timescales or under finite-temperature conditions, nuclear motion will introduce additional degrees of freedom that influence the electron dynamics.

Question 3: The calculations appear to be noiseless as Figure 4 shows exact mirror images. This gives faith in the numerical precision of the employed algorithm, but I wonder what deliberately adding some random noise (to simulate weak intermolecular interactions) could give. At what level of noise does the mirror symmetry disappear? This may tell something about the robustness of the effect and the chance to observe this in finite temperature experiments.

Response: Indeed, this is an interesting suggestion by the reviewer. In our opinion, the most meaningful way to introduce random noise is through the thermal occupation of molecular vibrational states. In this approach, the ions are selectively displaced according to an ensemble of molecular geometries sampled from the Boltzmann distribution at a given temperature. Previous studies have shown that such sampling can lead to rapid decoherence of electronic charge currents on a timescale of approximately 20 fs (M. Vacher *et al.*, *Faraday Discuss.* 194, 95 (2016); M. Vacher *et al.*, *Phys. Rev. A* 92, 040502 (2015); M. Lara-Astiaso *et al.*, *Faraday Discuss.* 194, 41 (2016)), although the exact extent depends strongly on the system and conditions. We anticipate that our observations would remain measurable within the first few femtoseconds, before quantum decoherence becomes significant. Furthermore, performing such a study would require considerable computational effort and would diverge substantially from the primary focus of the current work. Nevertheless, we will certainly take the reviewer's suggestion into account for future investigations.

Reviewer 2:

Report: The authors use quantum chemistry and quantum dynamics to simulate the excitation of a small fraction (≈ 3%) of an oriented achiral molecule from the ground state to excited states by means of a single ultrashort (few fs) right or left polarized vis/UV laser

pump pulse, cf. Figure 1(a) and Figure S4 and Table S1 of Supporting Information (SI). The ultrashort duration corresponds to a broad spectral width (> 1 eV) such that the laser excites at least two excited states, cf. Figures S1, S2, S3 of SI. At the end of the pulse, the molecule is thus prepared in a non-stationary superposition of at least three electronic states. The helicity of the laser pulse is transferred to corresponding electronic ring currents, cf. Figure 1(c). The related electronic densities are chiral, cf. Figure 1(b), with quasi-periodic chirality flips in the time domain of the period of the carrier frequency of the laser pulse (typically 1 fs). The electronic ring currents induce magnetic dipole moments which flip synchronously, cf. Figure 2. The authors suggest monitoring these magnetic dipole moments by means of an ultrafast (delta-type) probe pulse, cf. Figures 3 and 4, that means by time-resolved electronic dichroisms (TR-ECD), also called transient absorption dichroism spectroscopy. The authors consider the molecular model with the nuclei frozen in the experimental geometry of the electronic ground state, cf. Table S2 of SI. The resulting ring currents and the laser induced flips of the magnetic dipole moments persist for at least 100 fs, cf. Figure 2(a),(b). The corresponding Fourier transforms yields patterns which remind of high harmonic generation, cf. Figure 2(c). In SI, the authors also calculate corresponding time evolutions of the electric dipole moments, yielding similar time evolutions as well as high harmonic type patterns, cf. Figure S5 of SI. The underlying theory is outlined in Section S1 of the Supporting Information (SI). Applications are for oriented achiral furan (main text) as well as for oriented benzene and aniline (SI). The field of research on laser induced chirality flips in molecules is a rather young hot topic, cf. Refs. (8),(9). The authors add important progress to this rapidly evolving field: (i) excitation of at least two electronic states by a single ultrashort circularly polarized laser pulse (ii) evaluation of the laser-induced ring currents and induced magnetic moments (iii) monitoring signatures of the chirality flips by means of ultrafast pump probe spectroscopy, i. e. TR-ECD. On the way, they discover (iv) high harmonic type spectra of the induced magnetic dipoles as well as the induced electric dipoles. (v) The results are achieved by means of impressive advances of the fundamental theory and using the ReSpect program which have been developed by A. Rubio and his partners since 2009, cf. Refs. [1], [3], [6] – [8], [10],[11] of SI. The resume is that this is excellent work which adds enormous progress of the field of electronic chirality flips in achiral molecules. The authors are to be congratulated, and the J. Phys. Chem. Letters should be proud of publishing this fascinating work. Before publication, the authors should consider, however, the following list of fourteen Special items.

Question 1: Title and TOC Graphic: Please use consistent terminology. Interested non-experts would understand the acronym "TR-ECD" in the TOC Graphic more easily if in the

title, “transient absorption circular dichroism spectroscopy” is replaced by “time resolved electronic circular dichroism spectroscopy”, in accord with e.g. the Figure legends 4, S6.

Response: We agree with the reviewer and use consistent terminology throughout the manuscript, TOC and SI.
Changes: We replaced the text in the title and abstract “transient absorption circular dichroism spectroscopy” by “time-resolved electronic circular dichroism”.

Question 2: TOC Graphic and Figure 1(c) and Video: There are myriads of arrows which should illustrate the flux densities of the ring currents, but these arrows are exceedingly small so that it is impossible for me (and presumably for many readers) to extract the patterns of the fluxes. The authors should replace this worthless presentation of the flux densities with a clear one.

Response: The reviewer raises a valid point; however, we currently do not have a technology that would project the exact calculated current density into a smaller number of arrows while preserving the critical mirror symmetry between the left- and right-handed circularly polarized pump-induced cases. For this reason, we have opted to retain the original image, which in our opinion is interpretable in detail when viewed on a screen.

Question 3: The authors should remind the reader, not only in the main text but also in the Abstract and in the Conclusions, that they consider the scenario of molecules with frozen nuclei. In contrast, vibrations in the electronic ground and excited states may deplete the overlap of the representative vibrational wave functions, and this would cause decoherence of the phenomena which are discovered by the authors. These effects are discussed for electronic chirality flips in RbCs, cf. G. Liu et al, ChemPhysChem 2024, 25, e202400595.

Response: We agree with the reviewer and remind the reader about the frozen nuclei configuration in the Conclusion. However, we believe that the frozen nuclear configuration is already sufficiently highlighted in the Abstract.
Changes: in Conclusion: “... offering a powerful tool to study attosecond chiral dynamics in non-ionized regimes and <u>without changing achiral nuclear configuration”.</u>
In page 8, added the requested citation to G. Liu et al., *ChemPhysChem*, **25**, e202400595, (2024).

Question 4: The authors should point to the following caveat, in the Conclusion Section: Flips of induced magnetic moments and the resulting magnetic fields may arise from flips of chiral (this work) as well as from achiral electronic currents induced by circularly polarized laser pulses, see the discussion in I. Barth and J. Manz, “Quantum Switching of Magnetic Fields by Circularly Polarized Re-Optimized pi Laser Pulses: From One-Electron Atomic Ions to Molecules”, in “Progress in Ultrafast Intense Laser Science VI”, Springer Series in Chemical Physics, Vol 99, 21-44, eds K. Yamanouchi, A. D. Bandrauk and G. Gerber (Springer, Berlin, 2010). Turning the table, the results presented by the authors may be considered as signatures, but not necessarily as proof of “light-induced persistent electronic chirality in achiral molecules”.

Response: Agree, we added the following sentence in Conclusion:
Changes: “Therefore, flips of induced magnetic moments and the resulting magnetic fields may arise from flips of chiral (this work) as well as from achiral[58] electronic currents induced by circularly polarized laser pulses.”. Here, the reference 58 refers to the book chapter suggested by the reviewer.

Question 5: The authors should comment on the following discrepancy of the effects of single circularly polarized laser pulses. On the one hand, they show that these laser pulses may induce alternating ring currents which in turn induce flipping magnetic moments. On the other hand, these laser pulses may also induce uni-directional electronic ring currents without any flips, see e.g. Ref. (11) together with I. Barth and J. Manz, Angew. Chem. Int. Ed. 2006, 45, 2962 and G. Hermann et al, J. Phys. Chem. A 2016, 120, 5360. The carrier frequencies of the laser pulses for these “non-flipping” scenarios are resonant to excitations of selected doubly degenerate states, e.g. in benzene (... which is also considered by the authors, but with different scenario of the laser pulses, see below). In contrast, Fujimura and coworkers investigated excitations of two non-degenerate states in aromatic molecules by means of ultrashort linearly polarized laser pulses, and they discovered flips of the electronic ring currents, see e.g. H. Mineo, S.H. Lin, Y. Fujimura, Chem Phys. 2014, 422, 103 and earlier work cited therein. This discrepancy suggests a working hypothesis, namely a necessary (but not sufficient) condition for inducing flips of the ring currents and the induced magnetic fields is simultaneous excitations of at least two non-degenerate states. Gratifyingly, this condition is satisfied by the authors’ application of ultrashort laser pulses ~4 fs) with broad spectral width (> 1 eV).

Response: We agree with reviewer on this point, and the discrepancy has been addressed on page 8 by adding:

Changes: "It is important to note that the alternating ring currents and magnetic moments discussed here are induced by a single circularly polarized laser pulse that excites at least two non-degenerate states, as demonstrated in Section S2. In contrast, for molecules belonging to non-Abelian point groups, which possess sets of degenerate states, such laser pulses can induce unidirectional electronic ring currents without any reversals,[13-15] provided that the carrier frequencies of the laser pulses are resonant with the excitations of selected doubly degenerate states." Here, the references 13-15 refer to the papers suggested by the reviewer.

Question 6: Along with Refs. (3-7), the authors should quote pioneering work by Koch, Leibscher and coworkers, e.g. D. S. Tikhonov et al, Pump-probe spectroscopy of chiral vibrational dynamics, Sci. Adv. 2022, 8, eade0311 and M. Leibscher et al, Quantum control of rovibrational dynamics of chiral vibrational dynamics, PRA 2024, 109, 012810.

Changes: References added as (8) and (9).

Question 7: The authors should clarify the following discrepancy: In Section S1 of SI, they consider "an electric field with equal intensity for all frequencies and directions". The derivations rest on the validity of this assumption. Accordingly, "the final TR-ECD spectra were obtained from three independent pumpprobe simulations with the probe field oriented along three Cartesian directions." But the pump-field is uni-directional!

Response: This is not discrepancy, since the first sentence was extracted by reviewer from the text discussing a general ECD theory, whereas the second sentence was extracted from the text discussing the pump-probe simulation protocol. To make SI non-ambiguous, we have made the following:
Changes: We replaced the original text "The final TR-ECD spectra were obtained from three independent pump-probe simulations with the probe field oriented along three Cartesian directions." by "The final TRECD spectra were obtained from three independent pump-probe simulations with the unidirectional pumpfield and probe field oriented along three Cartesian directions."

Question 8: Can equation (14) in SI be simplified? (It contains the expression √sigma2/sigma).

Response: Yes, the simplification of Eq.(14) in SI was done.

Question 9: In Table S1 of SI, the authors should specify the maximum intensity of the pump and probe laser pulses (e.g. in a footnote, in units of W/cm2). The value for the pump pulse is given in the main text, but it will be helpful to list it also in Table S1 of SI.

Response: Table S1 has been updated accordingly.

Question 10: Table S2 of SI has the Cartesian coordinates for furan in the YZ-plane, in accordance with Figure 1(a). But for benzene and aniline, it is in the XY plane or almost in the XY plane. If the pump laser pulse propagates along Z, as illustrated in Figure 1(a), then the scenario for benzene and for aniline is entirely different from furan. In fact, the scenario for benzene would be analogous to the scenario of G. Hermann et al, J. Phys. Chem. A 2016, 120, 5360, and one would expect similar patterns of the ring currents, not entirely different ones. The authors should check the Cartesian coordinates in Table S2perhaps they need to be permutated from XYZ to YZX?

Response: We have carefully verified the Cartesian coordinates reported in Table S2 and confirm that they are correct. Furthermore, the reviewer is correct in noting that the relative orientation of the pump and molecule differs between the furan case (light propagates along Z within the molecular plane) and the aniline/benzene case (light propagates along Z perpendicular to the molecular plane). The comparison to Hermann et al., *J. Phys. Chem. A*, 120, 5360 (2016), is not directly applicable for the following reasons: (a) the authors employ linearly polarized light, (b) the light is aligned along the molecular plane (X-axis), and (c) the non-stationary state is manually constructed as a 50:50 superposition of the ground and first excited states of benzene. Collectively, these differences result in a substantially different induced charge density.

Changes: In order to notify the reader about the different relative orientation of the pump and molecule in SI, we added the following statement in Section S6 "Note that the relative orientation of the pump and molecule differs between the furan case (light propagates along Z within the molecular plane) and the aniline/benzene case (light propagates along Z perpendicular to the molecular plane)".

Question 11: Figures S1, S2, S3 of SI compare the frequency domain of the circularly polarized laser pulses with the molecular absorption spectra and the bright excitation energies. The authors should explain how they calculated these absorption spectra and

excitation energies. Else they should quote the original publications of these spectra and energies.

Response: The details have been added to Section S2.

Question 12: The chiral molecular-current analogue to high harmonic generation HHG which is illustrated in Figures 2(c) and S5(c) extends to rather high energies. For comparison, the authors should also list the ionization potentials IP. If the energies of the HHG type spectra exceed IP, then the authors should comment on the possibility that their laser pulses might destroy their molecules by ionization.

Response: The reviewer's comment is justified, and we have included the estimates of the ionization thresholds in SI as Table S3. However, within the current theoretical framework, the observed higher-order harmonics should be interpreted solely as signatures of intense nonlinear responses rather than molecular ionization.
Changes: Table S3 has been added in SI to indicate the estimated ionization thresholds. In addition, in the main text on page 9, we replaced the original statement "Contrarily, the chiral-current harmonics do not cause light emission, but tune the speed with which the molecular handedness can ultimately be controlled." by "The observed higher-order harmonics in the chiral current tune the speed with which the molecular handedness can ultimately be controlled, and here they should be interpreted solely as signatures of intense nonlinear responses rather than molecular ionization."

Question 13: Section S5 of SI points to two primary observations (i) and (ii) of the video. Please add another set of snapshots for slow-motion camera of one cycle of the chirality switch of the electronic densities and flux densities, after the end of the laser pulse.

Response: The video has been slowed.

Question 14: The last sentence of Section S3 of SI mentions "wavepacket ...relaxation due to decoherence, gradually evolving toward the ground-state electronic density." The authors should specify the mechanism of decoherence.

Response: The statement is now reformulated such that it explains our observation more accurately. Namely, the observed gradual increase in the fractional ground-state population after the end of pump pulse results from coherent dephasing, as the off-

diagonal terms in the density matrix begin to interfere destructively, reducing the strength of coherent oscillations in population. This gives the appearance of damping but does not involve any energy loss or dissipation. The system remains in a fully coherent, nonequilibrium state throughout the evolution. As the correction, we replace the original sentence by

Changes: in Section S3 "Following the pump pulse, the electronic wavepacket undergoes coherent dephasing, giving the appearance of relaxation toward the ground-state density due to destructive interference among its eigenstate components."

Reviewer 3:

Report: This is a fascinating and well-written manuscript on a potentially new spectroscopic phenomenon, namely the generation of persistent chiral electronic currents in oriented achiral molecules, which may provide a new route to controlled attosecond chiral dynamics. Through real-time TD-DFT simulations of furan (with benzene and aniline reported in the SI), the authors examine oscillations in the magnetic dipole moment and observe a set of absorptions at the harmonics of the carrier frequency of the external circularly polarized field.

The manuscript is clearly written with sufficient information (including the SI) for others to reproduce the work. The results are compelling, and the analysis is insightful. I strongly recommend publication in JPCL after the authors have addressed the following minor points:

Question 1: Eq. (1): Please explicitly tie the L/R designation to -/+ in the field.

Response: We have clarified the connection between the L/R (left/right) notation and the $\mp$ sign in Eq.
(1).

Changes: Below Eq. (1), we added the following clarification "where the negative and positive combinations correspond to left (L) and right (R) circularly polarized light, respectively."

Question 2: p.8: "Contrarily, the chiral-current harmonics do not cause light emission, but tune the speed with which the molecular handedness can ultimately be controlled." What

is the evidence that the harmonics do not cause light emission? What part of the simulation includes emission dynamics upon which this statement would be supported?

Response: The reviewer is right on the fact that our claim was unsupported and caused confusion. Therefore, we have reformulated our statement such that it reflects our observation more accurately and eliminates potential misunderstandings.

Changes: In the main text on page 9, we replaced the original statement "Contrarily, the chiral-current harmonics do not cause light emission, but tune the speed with which the molecular handedness can ultimately be controlled." by "The observed higher-order harmonics in the chiral current tune the speed with which the molecular handedness can ultimately be controlled, and here they should be interpreted solely as signatures of intense nonlinear responses rather than molecular ionization."

Question 3: p.11: "delegated" --> "relegated"

Response: Corrected.

Question 4: p.12: "this microscopic behaviors" --> "this microscopic behavior"

Response: Corrected.

<u>Editorial Comments:</u>

Question 1: Since your manuscript mentions different parts of your graphic, such as A and B, then the graphic/caption must clearly contain all mentioned parts.

Response: We have now ensured consistent labelling across all figures.

Question 2: The TOC graphic should fit in an area no larger than 3.25 in. × 1.75 in. (approx. 8.25 cm × 4.45 cm) and should have adequate resolution and clarity. Confirm that all text is legible at this size.

Response: The TOC graphics has been changed to match these requirements.
